# Outline of a Genome Navigation System Based on the Properties of GA-Sequences and Their Flanks

**DOI:** 10.1371/journal.pone.0004701

**Published:** 2009-03-09

**Authors:** Guenter Albrecht-Buehler

**Affiliations:** Department of Cell and Molecular Biology, Feinberg School of Medicine, Northwestern University, Chicago, Illinois, United States of America; Pasteur Institute, France

## Abstract

Introducing a new method to visualize large stretches of genomic DNA (see Appendix S1) the article reports that most GA-sequences [1] shared chains of tetra-GA-motifs and contained upstream poly(A)-segments. Although not integral parts of them, Alu-elements were found immediately upstream of all human and chimpanzee GA-sequences with an upstream poly(A)-segment. The article hypothesizes that genome navigation uses these properties of GA-sequences in the following way. (1) Poly(A) binding proteins interact with the upstream poly(A)-segments and arrange adjacent GA-sequences side-by-side (‘GA-ribbon’), while folding the intervening DNA sequences between them into loops (‘associated DNA-loops’). (2) Genome navigation uses the GA-ribbon as a search path for specific target genes that is up to 730-fold shorter than the full-length chromosome. (3) As to the specificity of the search, each molecule of a target protein is assumed to catalyze the formation of specific oligomers from a set of transcription factors that recognize tetra-GA-motifs. Their specific combinations of tetra-GA motifs are assumed to be present in the particular GA-sequence whose associated loop contains the gene for the target protein. As long as the target protein is abundant in the cell it produces sufficient numbers of such oligomers which bind to their specific GA-sequences and, thereby, inhibit locally the transcription of the target protein in the associated loop. However, if the amount of target protein drops below a certain threshold, the resultant reduction of specific oligomers leaves the corresponding GA-sequence ‘denuded’. In response, the associated DNA-loop releases its nucleosomes and allows transcription of the target protein to proceed. (4) The Alu-transcripts may help control the general background of protein synthesis proportional to the number of transcriptionally active associated loops, especially in stressed cells. (5) The model offers a new mechanism of co-regulation of protein synthesis based on the shared segments of different GA-sequences.

## Introducton

The importance of genome navigation in the case of the huge genomes of mammals and others can hardly be exaggerated. As pointed out in a previous article [1], even the most basic household function of mammalian cells require finding specific genes reproducibly and rapidly in the multi-billion base pair vastness of their genomes, especially during immune or stress responses. The often cited random diffusion of transcription factors and polymerases throughout the dense chromatin matrix hardly represents a navigation system with the required high level of accuracy and speed.

Equally important seems to be the necessity to understand possible failures of genome navigation. Even a ‘mild’ slow-down of the search mechanisms may cause numerous diseases by delaying the synthesis and/or turnover of vital gene products and moving them out of a required synchrony. Worse, even a small mutation in the direction-giving elements may cause the misdirection of the search mechanism. By sending large numbers of polymerases to the wrong targets such a mutation may produce diseases that have no single cause, but are the result of hundreds and thousands of improper gene expressions that may seem functionally unrelated and, thus, render it almost intractable. One wonders whether cancer or various dementias are diseases of this kind.

In a previous article I have suggested that pure GA-sequences may serve as sign posts of the genome navigation system [1]. These are sequences of 50–1300 bases consisting exclusively of G's and A's. Statistically speaking, their existence is extremely improbable. Yet, tens of thousands of such sequences are distributed throughout mammalian genomes. With the exception of 4 specific types, no two of them were identical. Although there is no doubt that pure GA sequences have all these properties, there is as yet no experimental evidence that they serve as sign posts of a genome navigation system, even though a number of observations in the field of heat shock seem to support the interpretation [1].

In view of the pivotal importance of our understanding of the way in which genomes navigate their own vastness, this article tries to expand further on the concept of a genome navigation system which is based on pure GA-sequences in order to advance it to a more testable state.

## Results

A list of definitions used in this article are attached at the end)

### 1. The genome pixel image (GPxI)

The aim of the present article to study the sequence architecture of GA-sequences and their genomic neighborhood requires detailed comparisons of thousands of very large DNA sequences in order to detect common patterns among them. Traditionally, this kind of task is solved by aligning them and by computing their homology, using one of the established algorithms such as the Needleman-Wunsch algorithms [2].

While such methods are both mathematically elegant and quantitative, they require considerable computing time and, more importantly, they often require some prior knowledge as to which DNA sequences should be examined in order to obtain meaningful results. Therefore, I introduce and apply here a novel method to represent DNA sequences. It turns relationships between sequences into visible patterns by representing the DNA sequences as gray-tone images called ‘genome pixel images’ (GPxI). The method is both sensitive and intuitive as it takes advantage of the exceptional ability of the human visual sense to detect patterns in images.

Briefly, the method assigns to the bases the following gray-tone values: A: black, G: white, C: dark gray and T: light gray (Fig. S1). This assignment is, of course, arbitrary, but must remain the same throughout. It transforms the consecutive bases of a DNA sequence into a continuous line of pixels with these gray values. Whenever the line of pixels reaches the edge of the image area, it wraps around like any other text would, and continues at the beginning of the next line immediately underneath (Fig. S2). The method detects relationships between sequences as patterns very sensitively (Fig. S3). For more details see Appendix S1.

### 2. The GPxI of the GA-complexes

In addition to the pure GA-sequences themselves I recorded also their 400 [b] large flanks in various chromosomes of humans, chimpanzees, rhesus monkey, mouse, and zebrafish. It should be noted that some of the GA-sequences and their flanks had to be omitted as they were duplications for the following reason. If 2 consecutive GA-sequences were closer together than the flank size of 400 [b], their flanks would overlap and, thus be recorded twice, at least in part. Therefore, *the flanks of all GA-sequences closer than 1 [Kb] were eliminated throughout this article*.

**Figure 1:**
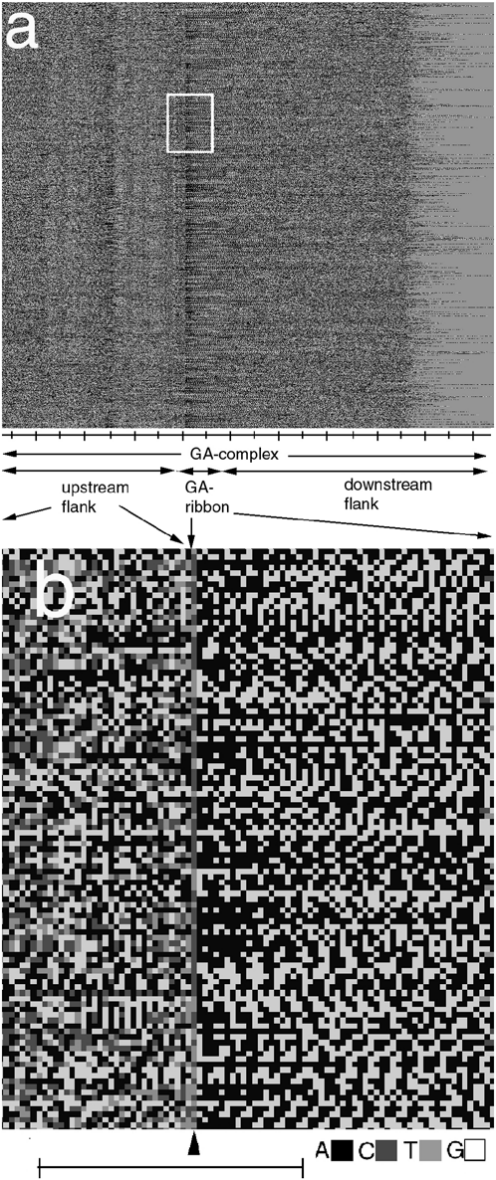
Typical appearance of the GPxI of the GA-complexes ( = upstream flank of 400 [b]+GA-sequence+downstream flank of 400 [b]) of human chromosomes. The GA-complexes are vertically aligned with the upstream ends of their GA-sequences. While the ends of all upstream flanks are automatically aligned, because they extend the same distance from the GA-sequences, the ends of the downstream flanks are not and appear frayed, as the length of each GA-sequence varies. The aligned GA-sequences in their natural order of occurrences in the chromosome are labeled as ‘GA-ribbon’. a. GPxI of the first 1000 GA-complexes of human chr.1 in their natural order of occurrence in the chromosome. Note the appearance of the ‘upstream stripes’ (see text) in the aligned upstream flanks and the predominantly black ( = poly-A) upstream beginnings of the aligned GA-sequences.(Scale: 50 [b]/division). b. Enlargement of the frame shown in panel a. Arrow points to the border between upstream flank and GA-sequence. By definition, it consists of T’’s or C's. (Scale: 50 bases).

The GPxI of the first 1,100 GA-complexes of human chr. 1 displayed in their natural order of occurrence are shown in Fig. 1a. The upstream ( = left hand) ends of all GA-complexes were aligned in the vertical direction, which automatically also aligned the upstream ends of the GA-sequences. In contrast, the downstream flanks were not aligned in this GPxI, because the lengths of the pure GA-sequence were variable [see 1], thus pushing the ends of the downstream flanks to variable positions.

There were 4 striking results of the depicted GPxI of the aligned GA-complexes.

1. The pure GA-sequences appeared to contain many non-random patterns.
2. Neighboring GA-sequences seemed to share many patterns as evidenced by the enhanced visibility of the patterns after alignment as in Fig. 1b.
3. Alternating stripes (‘upstream stripes’) appeared in the upstream flanks of certain primates.
4. In contrast, similarly aligned downstream flanks showed no pattern of any kind.

#### a. The tetra-GA motifs of pure GA-sequences

**Figure 2:**
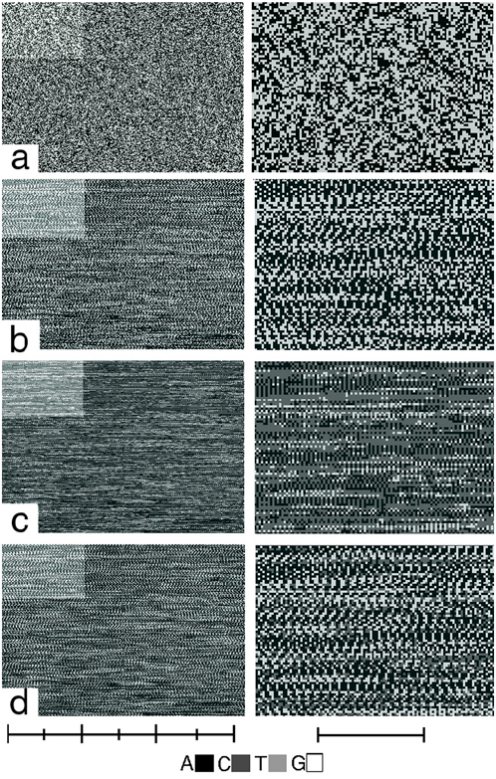
Predominance of tetra-GA motifs in the pure GA-sequences of human chr. 1 as demonstrated by the GPxI method. The highlighted field in the left hand panels are enlarged in the right hand panels.(Scales: 50[b]/division). a. The GPxI of a computer-constructed DNA file consisting of random sequences of G (white pixels) and A (black pixels). Therefore, no pixels with other gray-values are visible. The randomness is of the sequences is expressed by the lack of any detectable patterns. b. GPxI of the end-to-end concatenated pure GA-sequences of human chr. 1 shows clearly a number of patterns. Although different, they seem to share a periodicity of 4. c., d. Use of a modified Markham rotation [3] to demonstrate the prevalence of the 4-periodicity. In panel c the GPxI of panel b is superimposed on itself although frame shifted by 2 bases. The result is a rather featureless gray image. In panel d the applied frame shift is 4. The result is the almost identical re-appearance of the original GPxI, indicating that a frame-shift of 4 reinforces the prevalent patterns.

Concatenating end-to-end all 1667 pure GA-sequences of human chr.1 yielded the GPxI shown in Figure 2b. The comparison with a computer-constructed random GA-sequence file (Figure 2a), confirmed that the pure GA-sequences contain many repetitive patterns.

The period length of the common motifs can easily be determined by yet another application of the GPxI-method. Adopting the rationale of the so-called Markham rotation [3], one can superimpose pixel-by-pixel a particular GPxI with other GPxIs that were created by frame-shifts of 1,2,3, …[b] of the original sequence. Assume a motif has the size of N bases and forms strings of various lengths. Every time the original GPxI is superimposed with one that was frame shifted by N or an integral multiple of N, the images of the motif strings coincide and thus appear reinforced.

As illustrated in the GPxI of the pure GA-sequences of human chr.1 (Fig. 2b) frame shifts of 4, but not of 1, 2, and 3 reinforced the patterns, indicating that the prevalent repeated motifs of pure GA-sequences are tetra-GA motifs. These motifs were not only present, but constituted a significant part of the pure GA-sequences. Furthermore, the 4-fold patterns seem to repeat over several lines in the vertical direction of the GPxI, as if consecutive GA-sequences shared similar chains of tetra-GA motifs.

Many of the 16 different tetra-GA motifs (AAAA, AAAG, AAGA, AGAA, GAAA, GAAG, GGAA, AAGG, AGGA, AGAG, GAGA, GAGG, AGGG, GGAG, GGGA, GGGG) give rise to the same repetitive chains, provided one disregards the first 2 or 3 bases with which the chains begin. For example, chains of any of the 4 tetra-GA-motifs AAAG, AGAA, AAGA, and GAAA will generate essentially the same sequence …AAAGAAAGAAAGAAAGAAAG…. Only the beginning and ends of the chains may differ.

Similar considerations suggest that in addition to AAAG among the remaining tetra-GA-motifs only AAGG, AGAG, and GGGA were able to generate essentially different chains (AAAA and GGGG are excluded by definition of the pure GA-sequences). These tetra-GA-motifs occurred with different frequencies in the pure GA-sequences. Evaluating the 206,450 occurrences of tetra-GA motifs in the 19,139 pure GA-sequences of the entire human genome yielded the following probabilities of their occurrence: AAAG (10.4%), AAGG (7.1%), AGAG (5.1%), and GGGA (3%). Together all of the tetra-GA motifs made up 46–47% of the entire length of the pure GA-sequences of the human genome. The rest were individual sequences that guarantee the individuality of the GA-sequences [1].

#### b. The appearance of upstream stripes in the GPxIs of the GA-complexes of human and chimpanzee

A closer inspection of Fig. 1a suggests that the stripe patterns appeared upstream of a pure GA-sequence whenever its upstream end began with a certain stretch of poly(A) (i.e. with many black pixels). In order to test this conjecture, I extended the definition of GA-sequences to include more cases with poly(A) stretches.

At this point the reader is reminded that pure GA-sequences were defined as GA-sequences longer than 50 bases in order to *exclude* poly(A) and poly(G) sequences which, of course, fulfill trivially the definition of a GA-sequence, namely to contain no C's or T's [1]. Therefore, the inclusion of more poly(A) containing GA-sequences was achieved by simply easing the size restriction down to sizes of only 20 bases and longer. The resulting GA-sequences will be called ‘common’ GA-sequences in the following. By definition, the common GA-sequences included the pure ones.

**Figure 3:**
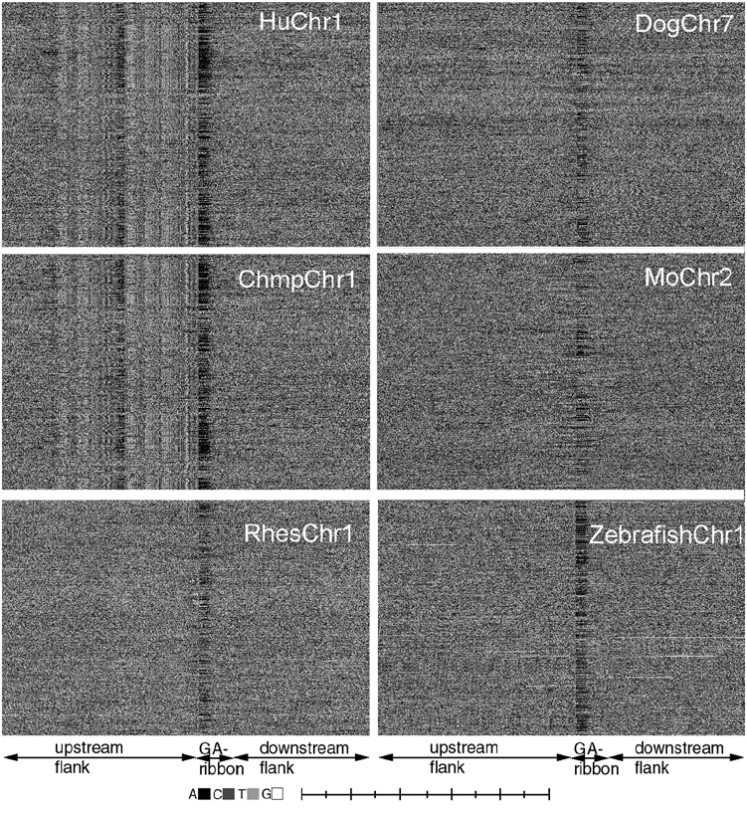
Architecture of the upstream flanks of selected chromosomes of various vertebrates. The GPxIs were obtained by aligning the upstream ends of the common GA-sequences in their natural order of occurrence in the chromosomes. It appears that only human and chimpanzee chromosomes express upstream stripes. However, the upstream stripes of human and chimpanzee were identical.(Scale: 50[b]/division).

Reducing the length restriction yielded a much increased number of GA-sequences. For example, human chromosome 1 contained 1667 pure GA-sequences and 19,513 common GA-sequences. As a result, the ribbon of GA-sequences became much darker in the GPxI and the upstream stripes became much more pronounced (Fig. 3).

Upstream stripes appeared in identical form in the GPxIs of the (common) GA-complexes of human chromosomes 1 (Fig. 3), 7 and X and even in the GPxIs of chimpanzee chromosomes (Fig. 3). In contrast, chromosomes of rhesus monkey, dog mouse and zebrafish showed no obvious patterns in the upstream flanks (Fig. 3).

**Figure 4:**
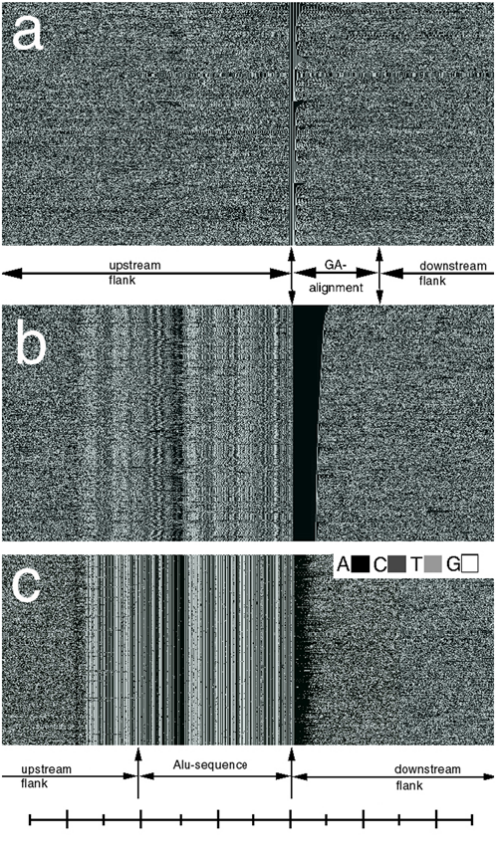
Identity between upstream stripes and Alu-sequences and their expression as a function of upstream poly(A)-segments of the GA-sequences. The GPxIs show portions of the GA-complexes of human chr.1 after sorting them by the decreasing size of poly(A)-segments at the upstream end of the GA-sequences. The aligned GA-sequences are labeled as ‘GA-alignment’ because they are not depicted in their natural order. (Scale: 50[b]/division). a. Absence of upstream stripes wherever the upstream ends of the GA-sequences contained no poly(A)-segments. b. Strong expression of upstream stripes where the GA-sequences ended in large upstream poly(A)-segments (black stretches). c. GPxI of the matches of the Alu-consensus sequence cited in the text and their 400 base large up- and down-stream flanks found in human chr.1. Note, the Alu-pattern extends upstream beyond the limit of the consensus sequences. Numerous point mutations can be seen as individual pixels that have a different gray value than the consensus pattern above and below. Furthermore, each Alu-sequences seems to terminate downstream in a stretch of black pixels, i.e. in a poly(A)-sequence.

The GPxIs generated from the common GA-sequences of human and chimpanzee chromosomes after re-ordering them by the size of their upstream poly(A)-segment confirmed that the upstream poly(A) stretches were required for the appearance of upstream stripes: Whenever the GA-sequences did not end in an upstream poly(A) motif, upstream stripes were not visible in the GA-complex, either (Fig. 4a). In contrast, when the GPxI of a GA-sequence displayed a predominantly black stretch, the upstream stripes were strongly expressed in its upstream flank (Fig. 4b).

They also demonstrated that the poly(A)-segments (depicted black in the GPxIs) were located almost exclusively at the upstream ends of the GA-sequences (see e.g. Fig. 3). In this way, the poly(A)-segments created a certain asymmetry and directionality of the GA-sequences, which may point to their role as markers for a reading direction of the GA-sequences.

Apparently, in exceptional cases GA-complexes can suffer inversions. After sorting the GA-complexes according to the poly(A) content of their downstream flanks, I found in human chr.7 a handful of GA-complexes whose upstream stripes were absent, but their exact mirror images appeared in the GA-sequences.

#### c. The identity between upstream stripes and Alu-sequences

In an unrelated study I searched the human chromosome 1 for the locations of Alu sequences. The search used the Alu-sequence of Def {4} as template and tolerated up to 10 point mutations at arbitrary locations for successful matches.

Once found, the matching sequences and their 400 [b] large up- and downstream flanks were recorded and used to generate the GPxI of the corresponding Alu-complexes (Fig 4c). Surprisingly, the upstream stripes of human and chimpanzee appeared identical to the stripe pattern of the Alu-sequences (Fig. 4b,c). A further surprise was the absence of any Alu-patterns in the upstream flanks of the GA-sequences of rhesus monkeys (Fig. 3), (or anywhere else in the rhesus genome), as Alu-sequences are generally believed to be shared by all primates.

## Discussion

### 1. A simple model of genome navigation

The following describes in broad strokes an outline of genome navigation that is consistent with the above findings. It offers details only when there were obvious objections to be met.

#### a. The need to concentrate the sign posts into a small space

If genomes, indeed, contain sign posts in the form of pure GA-sequences, it is rather obvious, what a genome navigation system should *not* do. Imagine that it would need to scan the entire genome in order to find a particular sign post, which subsequently would guide it to the desired target genes. This mechanism would offer very little advantage over no navigation mechanism at all. After all, instead of crawling along billions of bases to find a specific gene, the search mechanism would have to crawl along the same billions of bases in order to find first the appropriate sign post. Obviously, it would be much more efficient, if all sign posts were concentrated in a small space, so that the search mechanism could rapidly leap from one sign post to the next.

#### b. The topology of the side-by-side alignment of GA-sequences

**Figure 5:**
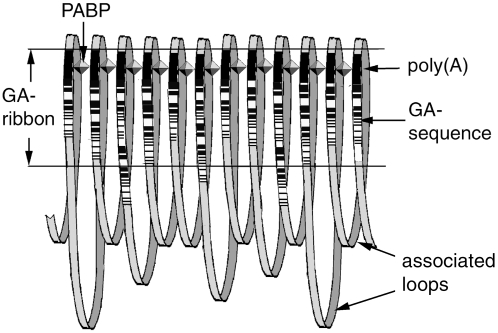
Side-by-side alignment of consecutive GA-sequences by poly(A) binding proteins (PABP). The GA-sequences (black and white striped segments) are assumed to be sign posts for a searching mechanism that uses their upstream poly(A)-segments (black stretches) as markers for the reading direction and as binding sites for PABPs that link them side-by-side. The intervening stretches of genomic DNA have variable sizes and loop around to the next GA-sequence. The parallel arrangement of GA-sequences is called the ‘GA-ribbon’. The GA-sequences are assumed to be associated with DNA binding proteins that are specific for tetra-GA motifs (not shown; see Fig. 7).

**Figure 6:**
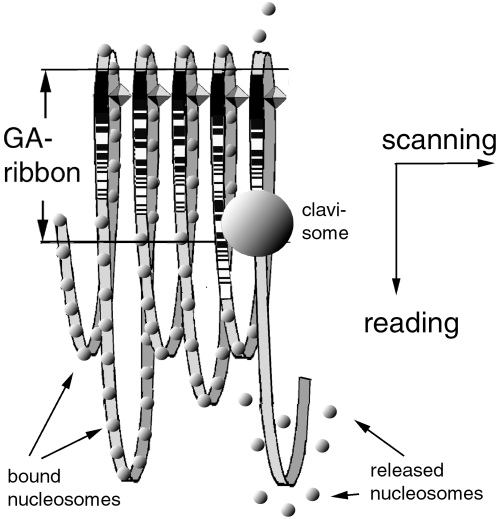
Outline of a chromatin model that supports a fast genome navigation system. By leaping from one GA-sequence to the next along the GA-ribbon in the scanning direction while ‘reading’ the information encoded in the proteins bound to the GA sequence in the reading direction, the postulated ‘clavisomes’ (searching complexes) can efficiently find the appropriate GA-sequence on a more than 700-fold shorter search path than by crawling along the various size loops of genomic DNA. After a clavisome found its target GA-sequence and interacted with it, the nucleosomes in the associated loop are released, and the specific coding sequences in the loop are exposed to the transcription mechanisms.

Since all pure GA-sequences of a chromosome are lined up in tandem on the same DNA strand, there is essentially only one non-disruptive way of forcing all of them into a small space, namely by placing the pure GA-sequences side-by-side while folding the intervening stretches of DNA between them into loops (See Fig. 5, Fig. 6).

#### c. The role of the upstream poly(A) stretches as binding sites for linker molecules

A side-by-side arrangement of consecutive GA-sequences requires one or more species of linker molecules which are capable of binding to consecutive GA-sequences and to each other. Therefore, all GA-sequences should, their overall individuality [1] notwithstanding, contain or be flanked by a common binding segment for these universal linker molecules. Based on the above results, the poly(A) stretches at the upstream end of the common GA-sequences are the most obvious candidates for such common binding sites for linker molecules.

In this case, it would not be difficult to find the corresponding poly(A) binding proteins that could serve as the linker molecules. Although primarily known for their interaction with the 3′ poly(A) tails of mRNAs, in many cases their binding specificity does not distinguish unambiguously between poly(A) and poly(dA). Especially the protein known as PABPN1 was found to enter the nucleus, to be required for transcription [4], [5], and to shuttle between nucleus and cytoplasm [6]. More importantly, it not only binds to poly(A) sequences, it appears to bind to itself, as it forms nuclear aggregates even in the absence of mRNA [4]. In this way, it would be able to bind two poly(A) sequences together in a poly(A)-PABPN1-PABPN1-poly(A) complex. Hypothesizing, therefore, that PABPN1, or similar nuclear poly(A) binding proteins align GA-complexes with their upstream poly(A)-segments, one may arrive at a basic topology of chromatin that would support a fast genome search and navigation mechanism depicted as depicted in Fig. 5.

The poly(A) binding protein(s) may have an additional role. GA-sequences as sign posts are likely to contain information for the searching mechanism, which may require a particular reading direction. Considering that poly(A) tails generate a reading asymmetry in mRNAs, it is tempting to think of the poly(A)-segments as markers for the reading starts, as they occur almost exclusively at the upstream end of the GA-sequences.

#### d. The reduction of the search path

Since the distance between consecutive GA-complexes is not constant, the parallel arrangement of GA-sequences would create a kind of ‘ribbon’ with different size loops between them (Figure 5). In this way, each chromosome is divided into 2 domains, the ribbon of the GA-sequences (non-coding) and all the rest (including all genes). In other words, all genes are located on one or the other loop. The variable sizes of the loops accommodate variable numbers and sizes of genes.

This ribbon may be considered a ‘macro-insulator’ [see 7] for an entire chromosome, although the present article presented no evidence that the associated DNA-loops between GA-sequences are transcriptional independent. Yet, in support of this notion, it appears that the known insulators in Drosophila melanogaster contain binding sites for the GAGA-factor [cited from 8], suggesting that they may be related to the GA-sequences.

Searching along this ribbon instead of the entire chromosome could shorten considerably the search path for genome navigation. Consider the following rough estimate! Human chr. 1 has a length of 238 Mb and contains 19513 common GA-sequences with an average distance between consecutive GA-sequences of 12.2 Kb. At a distance of 0.3 nm per base pair, the average loop between consecutive GA-sequences would therefore measure 3655 nm. If there was a base-by-base search mechanism it would have to crawl along this distance in order to move from one GA-sequence to the next. On the other hand, searching along the ribbon of parallel arranged GA-sequences would shorten the distance to the next GA-sequence to 1 diameter of the double helix (2 nm) and, maybe, the diameter of a linker protein (e.g. 3 nm). Thus, instead crawling for 3655 nm along a loop, the search mechanism could leap to the next sign post by moving only 5 nm, corresponding to a 730-fold shortening of the search path.

#### e. The GA-ribbon as an architectural feature of chromatin

Another kind of ribbon, namely the GA-ribbon had been introduced earlier (e.g. see Fig. 1a), in the GPxIs of GA-complexes. At that time, it was merely a visual consequence of sequence alignment in the GPxIs of chromosome segments. Now, based on the postulate of minimizing search paths, I suggest that the GA-ribbon may actually reflect a certain reality of chromatin architecture. In other words, if one could flatten out chromatin and stain the different bases with 4 different gray-tone probes, the resulting microscopic image may look similar to the GPxI of Fig. 1a.

It should be noted that the concept of the GA-ribbon is ultimately a topological one. If it were stained with appropriate probes, its actual appearance inside the nucleus does not have to resemble a straight ribbon. On the contrary, in actuality it may well be rolled up into a ball or a tube with the various associated DNA loops pointing to the outside. A number of other topologically equivalent shapes are equally well conceivable, and are not excluded here.

#### f. The chromatinization of GA-ribbon and associated loops

Of course, in reality the various loops of DNA will have to be associated with nucleosomes (Figure 6). The average size loop of 12.2 Kb is large enough to accommodate roughly 50–60 nucleosomes, or about one 30-nm fiber [9]. The variable lengths of the 30 nm fibers would be consistent with the variable length of the loops between adjacent GA-complexes.

**Figure 7:**
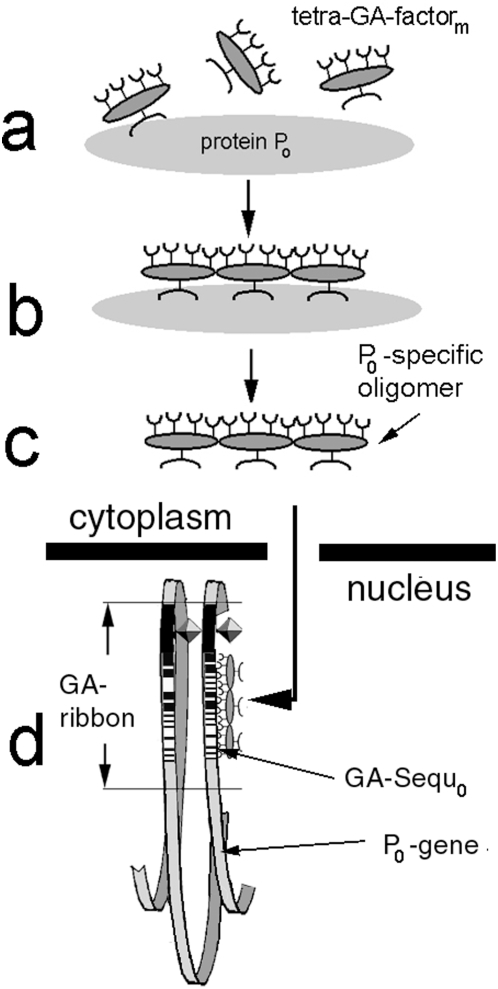
Assumed linkage between the cellular demand for protein P_0_ and the accessibility of the particular GA-sequence GA-Sequ_0_ which connects to the loop containing the P_0_ gene (see text). a. As long as the cellular protein P_0_ is available in sufficient quantities (i.e. there is no demand for P_0_), one or more of the 16 conceivable tetra-GA specific transcription factors tetra-GA-factor_m_ can bind to it at its specific binding sites. b. The bound tetra-GA-factor_m_ molecules form a P_0_-specific oligomer, [tetra-GA-factor_m1_] [tetra-GA-factor_m2_]… [tetra-GA-factor_mN_]. c. The P_0_-specific oligomers are released from the P_0_ molecule and enter the nucleus. d. They bind to the characteristic chains of the tetra-GA-motifs of GA-Sequ_0_ and prevent clavisomes from interacting with it. Conversely, if the cytoplasmic levels of P_0_ drop below a certain threshold (i.e. there is high demand for P_0_), no more P_0_-specific oligomers are formed to block GA-Sequ_0_. As a result, clavisomes are able to initiate the transcription of the genes for P_0_ in the associated loop of GA-Sequ_0_.

The GA-sequences whose parallel arrangement gives rise to the GA-ribbon would hardly exist as naked DNA for long, either. More likely they are associated with GA-specific transcription factors and other GA-specific DNA-binding proteins. In view of the reported prevalence of tetra-GA motifs in GA-sequences one would expect that these DNA-binding proteins have preferences for tetra-GA motifs such as the GAGA-factor [10], [11], HSF1 [12] and others (see below and Fig. 7d).

#### g. The postulate of a search mechanism (‘clavisomes’)

If one adopts the view that genomes contain sign posts arranged into much shortened search paths, it is consistent to postulate also that ‘something’ exists that searches this path. This hypothetical searching complex must (a) find the specific GA-sequence, GA-Sequ_0_ (see Def {7}), that belongs to the associated loop containing the gene of the target protein P_0_ (see Def {6}) and (b) interact with it in order to initiate transcription in the associated loop(s). Much may already be known about this entity, albeit possibly under different names. While there is no evidence that it exists in the form of a nuclear particle, for the sake of simplicity it will be treated as such and called a ‘clavisome’ in the following (from *lat*. clavis = key) as it ‘unlocks’ a segment of chromatin (see Def. {5}).

#### h. The hypothetical initiation of transcription by clavisomes

How can clavisomes initiate the transcription of a specific protein P_0_ in response to its demand by the cell? Several reports in the literature suggested that the activation of genes is accompanied by loosening or even breaking the association between DNA and nucleosomes [for references see 9]; [specifically 13] [and 14]. Thus it seems conceivable that the interaction between a clavisome and GA-Sequ_0_ leads to the release of the nucleosomes in the associated loop, thus exposing the coding sequences for P_0_ within the loop to the mechanisms of transcription. This step is depicted in the model of Figure 6.

#### i. The hypothetical recognition of target GA-sequences by clavisomes

Of course, the above hypothesis begs the question how clavisomes, upon a cellular demand for P_0_, can distinguish its particular GA-Sequ_0_ from all other GA-sequences. Assume, that each cellular protein P_0_ is able to interact with a certain number of hypothetical transcription factors, tetra-GA-factors_m_ (see Def {8}), which are specific for binding one of the 16 different tetra-GA motifs (Fig. 7a,b). The interaction may catalyze the formation of a P_0_-specific oligomer (see Def {9}) of these transcription factors, which is subsequently released from the P_0_ molecule and enters the nucleus (Fig. 7c). There it binds to the characteristic chains of the tetra-GA-motifs of GA-Sequ_0_ (Fig. 7d) and prevents clavisomes from interacting with GA-Sequ_0_. As a result, no new transcripts of P_0_ will be made as long as the cytoplasmic levels of P_0_ remain sufficiently high to produce a steady stream of the P_0_-specific oligomers. However, if the cytoplasmic levels of P_0_ drop below a certain threshold, GA-Sequ_0_ would become ‘denuded’ and allow clavisomes to initiate the transcription of the genes for P_0_ in the associated loop of GA-Sequ_0_.

The binding of the tetra-GA-factors_m_ by each cellular protein does not have to be direct. The proposed regulation of the P_0_ synthesis may happen indirectly via another protein that does bind these transcription factors. As to the case of secreted proteins, the model predicts that their transcription is only turned on if their steady state levels in the cytoplasm is depleted.

There are precedents for major aspects of the above scheme. For example, in Drosophila the GAGA-factor, which can be viewed as one of the 16 tetra-GA-factor_m_ molecules, is required to bind to GAGA motifs in certain GA-rich sequences before the transcription of heat shock proteins is initiated [11]. Another example may be the case of human HSF1, a GA-specific transcription factor with a pentameric consensus sequence of nGAAn which has to form a trimer in the cytoplasm in response to stress situations before it can initiate the transcription of heat shock proteins [12].

More generally speaking, it is not hard to imagine how natural selection, starting with some crude linkages between GA-motifs and GA-rich sequences, over time could have selected for gene products P_0_ with binding domains for the same combination of tetra-GA-factors_m_ as were contained in their corresponding GA-Sequ_0_.

#### j. Expected properties of the P_0_-specific oligomers

The formation of the P_0_-specific oligomers must not be a mere concatenation, nor must their inhibition of clavisomes be merely the result of their binding to the tetra-GA-motifs of the GA-sequences. After all, the individual tetra-GA-factors_m_ were assumed to bind these motifs, too. Unless prevented from entering the nucleus, the tetra-GA-factors_m_ should coat all the GA-sequences and, thus, stop transcription permanently. Therefore, the formation of P_0_-specific oligomers and their binding to the GA-sequences must involve additions and/or modifications of their component tetra-GA-factors_m_.

The postulated P_0_-specific oligomers need not be larger than pentomers in order to distinguish between more than 1 million different protein species P_0_ because 16^5^ = 1,048,576. Still, the regulation of the protein synthesis of P_0_ would probably require the binding of several P_0_-specific oligomers to GA-Sequ_0_. Otherwise, the expression of P_0_ would occur in a rather abrupt all-or-none fashion.

Multiple interactions between P_0_-specific oligomers and GA-Sequ_0_ would also be required if the P_0_-specific oligomers were composed of fewer than 5 monomers. In this case single tetra-, tri-, or dimers could not contain enough information to specify the target protein unambiguously among hundreds of thousand other candidates.

#### k. The potential for co-regulation of gene expression due to the similarity of neighboring GA-sequences

Frequently, the genes of co-regulated proteins are located in close proximity to each other on the genome [15]. The above outline of genome navigation offers several reasons to explain this finding.

In the first place, it assumes tacitly that all genes that are contained in an associated loop are expressed together. In this sense it provides a simplistic mechanism of co-regulation of gene expression.

Furthermore, many neighboring GA-sequences shared partly identical chains of tetra-GA motifs, as shown in Fig. 1b and 2b which depicted the GA-sequences of human chr.1 in their natural order of occurrence. Therefore, many P_0_-specific oligomers that bind to GA-Sequ_0_ may also bind to neighboring GA-sequences and, thus, influence the regulation of other gene products encoded in the adjacent associated loops.

This mechanism may even apply to genes of co-regulated proteins on different chromosomes. If their GA-sequences share similar chains of tetra-GA motifs their specific oligomers can cross-react with each other's Ga-sequences regardless of the distance between their loci and, thus, rise and fall together in their expression.

### 2. The close association between Alu-elements, poly(A)-sequences and GA-sequence in some primates

As reported in this article, the upstream ends of many GA-sequences were poly(A)-sequences. In human and chimpanzee genomes this has a peculiar consequence, because these genomes contain millions of Alu-sequences, which seem to be located upstream of poly(A) sequences (see e.g. [16] and Fig. 4c). Indeed, in humans and chimpanzees I also found Alu-sequence upstream of all the GA-sequences that terminated upstream in a poly(A)-sequence (Fig. 4b).

Of course, this association between GA-sequences and Alu-sequences in human and chimpanzee genomes may have been merely a consequence of a poly(A)-dependent insertion mechanism of Alu-sequences. However, as argued below, genome navigation may also benefit from these insertions.

Being retro-transposons, even a few Alu-elements could spell disaster for any genome, as they could exponentially replicate and re-insert and, thus, fragment the genome in the process. Apparently, humans and chimpanzee genomes have learned in time to inhibit the transcription of Alu-elements to a manageable level [16]. Assuming that this mechanism of suppression spills over to their flanking segments, the insertion of Alu-elements directly upstream of GA-sequences may have offered these primate genomes an added level of precise suppression of the transcription of GA-sequences, even while they are de-repressed, consistent with their function as sign posts.

Furthermore, one can imagine that a clavisome is able to initiate the transcription of the adjacent (previous or subsequent) Alu-sequence, once it has opened up a specific loop for transcription (Fig. 6). This action [16] may offer yet another advantage.

It is well known that Alu-transcripts have a substantial influence on the translational regulation of protein synthesis, especially in stressed cells [17], [18], [19]. Thus, if their transcription is contingent upon the opening of the target loops, they may afford the genome navigation mechanisms a swift and guaranteed handle on the control of ongoing gene expressions. This may be needed especially under stress situations. Indeed, it has been shown that genotoxic stress initiates a massive transcription of Alu-elements [20].

In view of the latter possibility the specific placement of Alu-sequences in the upstream flanks of GA-sequences may even offer a certain quantitative control of background protein synthesis: If each GA-sequence has its own upstream Alu-sequence, the genome navigation mechanism(s) may produce proportionally as many copies of Alu-transcripts as they opened loops for transcription.

Other organisms may not use the same mechanism(s) because their SINE or LINE sequences may not have the same effects on protein synthesis. Furthermore, humans and chimpanzees are among the most recent species. Therefore, one may consider the presence of Alu-sequences in the upstream flanks of their genomes as a very recent acquisition in the evolution of the genome navigation systems.

### 3. A note about the GA-sequences of Drosophila melanogaster

Finally, I should explain my frequent use of examples from Drosophila melanogaster, even though it is obviously not a vertebrate. In the first place, a number of reports in this field mention GA-related observations that seem to point to fundamental principles of genome navigation. Furthermore, I hope that a number of crucial predictions of the model presented here can be tested in Drosophila genomes. It may also be possible to find the corresponding results in vertebrate genomes. Nevertheless, there are major differences between Drosophila and vertebrate genomes that may warrant different tests and approaches.

For example, the GAGA-factor was discovered in the field of Drosophila and plays a major role there. Indeed, as shown in Appendix S2, the common GA-sequences of Drosophila contain predominantly chains of the GAGA motif (see Appendix S2: list of GA-sequences and Fig. S4b, area ‘3’). In contrast, the many other tetra-GA-motifs which occur frequently in vertebrate genomes exist in only very small numbers in Drosophila. Therefore, vertebrates may express many other tetra-GA-motif-factors that are still to be discovered and whose function needs to be explored. Furthermore, although the GA-ribbon of Drosophila chromosomes may appear similar to that of vertebrates (Appendix S2: Fig. S4a), the GA-sequences are much shorter (Appendix S2: list of GA-sequences) and may require very different kinds of clavisomes to interact with them.

### Definitions

The following definitions were used in this article.

Def {1} **GA-complex** = Concatenated DNA sequence consisting of an upstream flank of 400 [b], the (pure or common) GA-sequence, and a downstream flank of 400 [b].

Def {2a} **GA-ribbon** = portion of the GPxI that contains the aligned GA-sequences in their natural order of occurrence in the chromosome.

Def {2a} **GA-alignments** = portion of the GPxI that contains the aligned GA-sequences not in their natural order, but re-ordered by a certain logical criterion (e.g. by the size of poly(A) sequences in the upstream end of the GA-sequences, etc. ).

Def {3a} **pure GA-sequence** = DNA sequence consisting exclusively of G's and A's for a length>50 [b]. It excludes most poly(A) and ploy(G) sequences

Def {3b} **common GA-sequence** = DNA sequence consisting exclusively of G's and A's for a length>20 [b].

Def {4} **Alu test sequ**.: (NT_025741.14|Hs6_25897; Human chr.6, 55141462: 55141641) AGGAGATCGAGACCATCCTGGCTAACACGGTGAAACCCCGTCTCTACTAAAAATACAAAAAATTAGCCGGGCGTGGTGGCGGGCGCCTGTAGTCCCAGCTACTCGGGAGGCTGAGGCAGGAGAATGGCGTGAACCCGGGAGGCGGAGCTTGCAGTGAGCCGAGATCGCGCCACTGCACTCCAGCCTGGGCGACAGAGCGAGACTCCGTCT.

Def {5} **‘Clavisomes’:** Hypothetical nuclear particles that contain the necessary molecular components to

- interact with the specific GA-binding proteins which cover the tetra-GA motifs and other motifs of the GA-sequences in order to recognize a specific target GA-sequence, and to
- release the nucleosomes from the loop associated with the target GA-sequences, and to
- initiate and support the transcription of the genes contained in the associated loop

Def {6} **P_0_**: A specific gene product, demanded by the cell.

Def {7} **GA-Sequ_0_**: The particular GA-sequence whose associated loop contains the coding sequence for P**_0_**.

Def {8} **tetra-GA-factor_m_**: The m^th^ of 16 conceivable transcription factors that are specific for 16 tetra-GA-motifs, AAAA, AAAG, AAGA, AAGG, AGAA, AGAG, AGGA, AGGG, GAAA, GAAG, GAGA, GAGG, GGAA, GGAG, GGGA, GGGG. The well-known GAGA-factor [11] may be one of them.

Def {9} **P_0_-specific oligomer:** The oligomer [tetra-GA-factor**_m1_**] [tetra-GA-factor**_m2_**]… [tetra-GA-factor**_mN_**] formed from N>4 tetra-GA-factor molecules which are capable of binding to **P_0_**. The P_0_-specific oligomer is assumed to bind to GA-Sequ**_0_** and inhibits the access of clavisomes to the associated loop.

## Materials and Methods

The genome sequences of human, chimpanzee, mouse, dog, zebrafish, and Drosophila melanogaster were obtained from the UCSC site. The Alu-sequence was derived from the NCBI site.

The analysis program, “GA_dnaorg.exe”, was written by G.A.-B. using Visual C++ (Microsoft, Redmond, WA).

## Supporting Information

Figure S1 Basic principle of the ‘genome pixel image’ (GPxI) method. (Scale: 50(b)/division) a. Assignment of a pixel value to each base. b. Creation of a pixel image by writing the sequence of a DNA file from left to right and top to bottom while expressing each base as a single pixel with the assigned gray-value. Whenever the pixel line has reached the edge of the image ( = GPxI-width), it wraps around and continues on the left and 1 pixel diameter down. The GPxI shown in panel b represents a computer-constructed, random DNA file.
(1.87 MB TIF)

Figure S2 GPxI of the first 150 Kb of the human X chromosome (Un-sequenced portions are omitted). (Scales: 50(b)/division) a. The appearance of several pseudo-repetitive sequences as various, seemingly repetitive patterns. The appearance of identical repetition vanishes with increasing magnification of the GP demonstrating the power of the human visual sense to still detect rules and relationships between DNA sequences after after mutations and variations have obliterated them to a large degree.. b. Enlargement of the portion of the GPxI within the black frame in panel a. c. Enlargement of the portion of the GPxI within the black frame in panel b.
(4.82 MB TIF)

Figure S3 Effect of GPxI-width on pattern appearance and recognition on a portion of the GPxI of Figure 2. The numbers 1,2,and 3 indicate the same domains on each panel. Enlargments of these domains are shown on the right hand side.(Scale: 50(b)/division) a. GPxI-width = 610 (b). The pattern at ‘1’ turns vertical but, as shown by the enlargement, contains deviations in the form of 2 shifts ( = insertions) and single deviant pixels ( = point mutations). b. GPxI-width = 568 (b). The domains ‘2’ and ‘3’ appear almost random. c. GPxI-width = 551 (b). Domain ‘2’ shows a clear periodicity with few deviations. Domain ‘3’ shows pseudo-repetitive patterns.
(0.85 MB TIF)

Figure S4 GPxI of the first 492 GA-complexes of Drosophila melanogaster chr.X. (Scale: 50 (b)/division) a. GPxI of the GA-complexes in their natural order aligned by the upstream start of the common GA-sequences. b. GPxI of the same GA-complexes sorted alphabetically. The right-hand insets show the 5× enlarged areas labeled 1, 2, and 3. 1: pure poly(A) GA-sequences. 2: poly(A) sequences ending in G. 3: one of the many examples of poly(GA) sequences.
(9.89 MB TIF)

Appendix S1 The genome pixel image (GPxI). I introduce and apply here a novel method to represent DNA sequences. It turns relationships between sequences into visible patterns by representing the DNA sequences as gray-tone images called ‘genome pixel images’ (GPxI). The method is both sensitive and intuitive as it takes advantage of the exceptional ability of the human visual sense to detect patterns in images.
(0.03 MB DOC)

Appendix S2 GPxI of the common GA-sequences of Drosophila melanogaster, chr.X. Many of the supporting experiments in this article about vertebrate genomes were quoted from data about this non-vertebrate organism. In order to facilitate the comparison for experts in the field of Drosophila, the Appendix shows the GPxI of the X chromosome and the complete list of common GA-sequences.
(0.44 MB DOC)
