# Supplementary figures and images for: Outline of a Genome Navigation System Based on the Properties of GA-Sequences and Their Flanks
Source: PLoS One. 2009 Mar 9;4(3):e4701. doi: 10.1371/journal.pone.0004701 (PMC2651618; doi:10.1371/journal.pone.0004701)

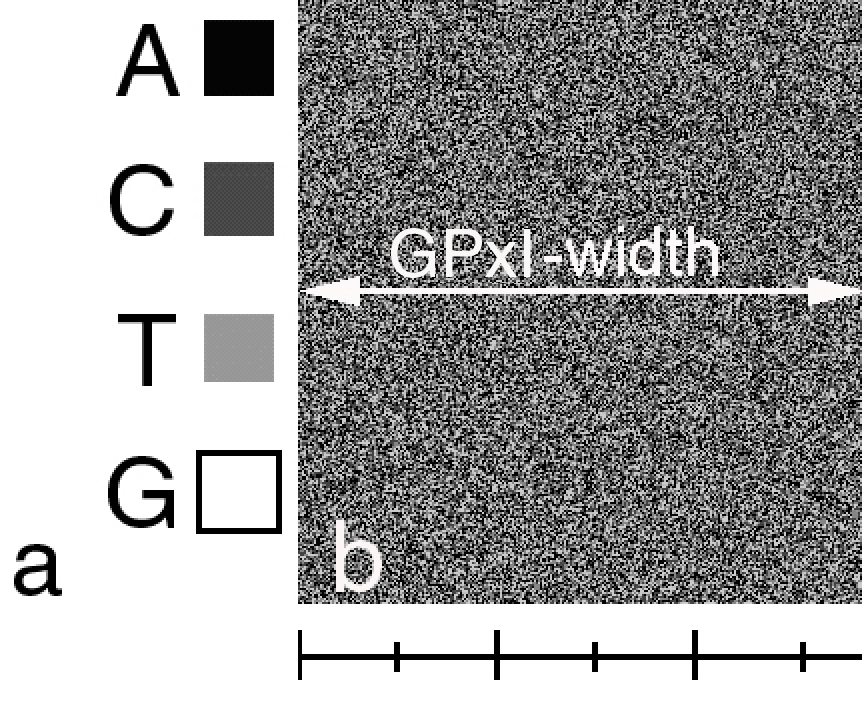

Supplement: Figure S1 — Basic principle of the ‘genome pixel image’ (GPxI) method. (Scale: 50(b)/division) a. Assignment of a pixel value to each base. b. Creation of a pixel image by writing the sequence of a DNA file from left to right and top to bottom while expressing each base as a single pixel with the assigned gray-value. Whenever the pixel line has reached the edge of the image ( = GPxI-width), it wraps around and continues on the left and 1 pixel diameter down. The GPxI shown in panel b represents a computer-constructed, random DNA file. (1.87 MB TIF) [file pone.0004701.s001.tif]

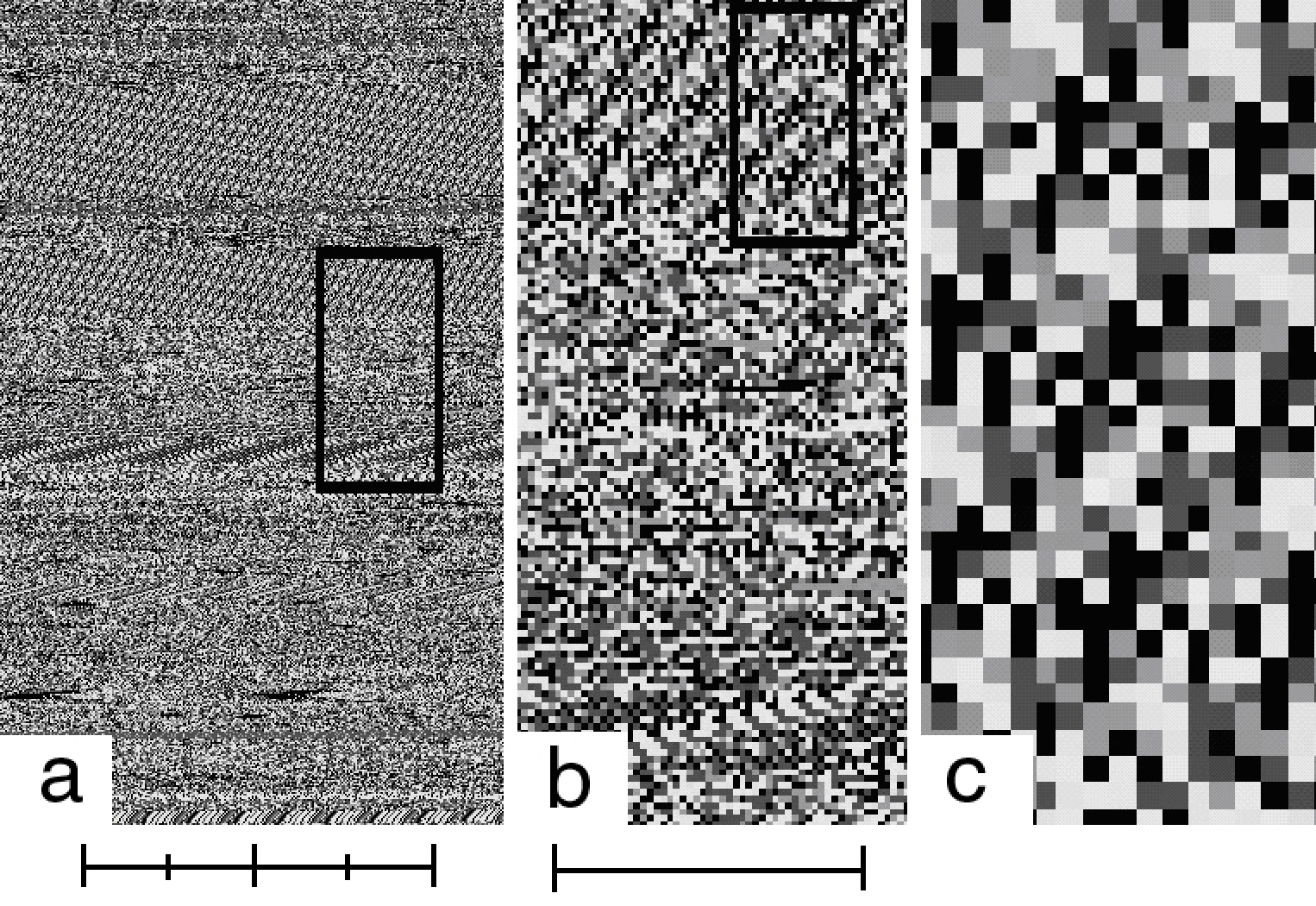

Supplement: Figure S2 — GPxI of the first 150 Kb of the human X chromosome (Un-sequenced portions are omitted). (Scales: 50(b)/division) a. The appearance of several pseudo-repetitive sequences as various, seemingly repetitive patterns. The appearance of identical repetition vanishes with increasing magnification of the GP demonstrating the power of the human visual sense to still detect rules and relationships between DNA sequences after after mutations and variations have obliterated them to a large degree.. b. Enlargement of the portion of the GPxI within the black frame in panel a. c. Enlargement of the portion of the GPxI within the black frame in panel b. (4.82 MB TIF) [file pone.0004701.s002.tif]

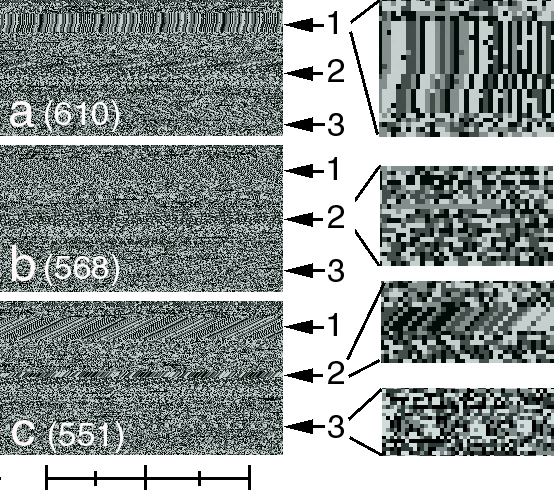

Supplement: Figure S3 — Effect of GPxI-width on pattern appearance and recognition on a portion of the GPxI of Figure 2. The numbers 1,2,and 3 indicate the same domains on each panel. Enlargments of these domains are shown on the right hand side.(Scale: 50(b)/division) a. GPxI-width = 610 (b). The pattern at ‘1’ turns vertical but, as shown by the enlargement, contains deviations in the form of 2 shifts ( = insertions) and single deviant pixels ( = point mutations). b. GPxI-width = 568 (b). The domains ‘2’ and ‘3’ appear almost random. c. GPxI-width = 551 (b). Domain ‘2’ shows a clear periodicity with few deviations. Domain ‘3’ shows pseudo-repetitive patterns. (0.85 MB TIF) [file pone.0004701.s003.tif]

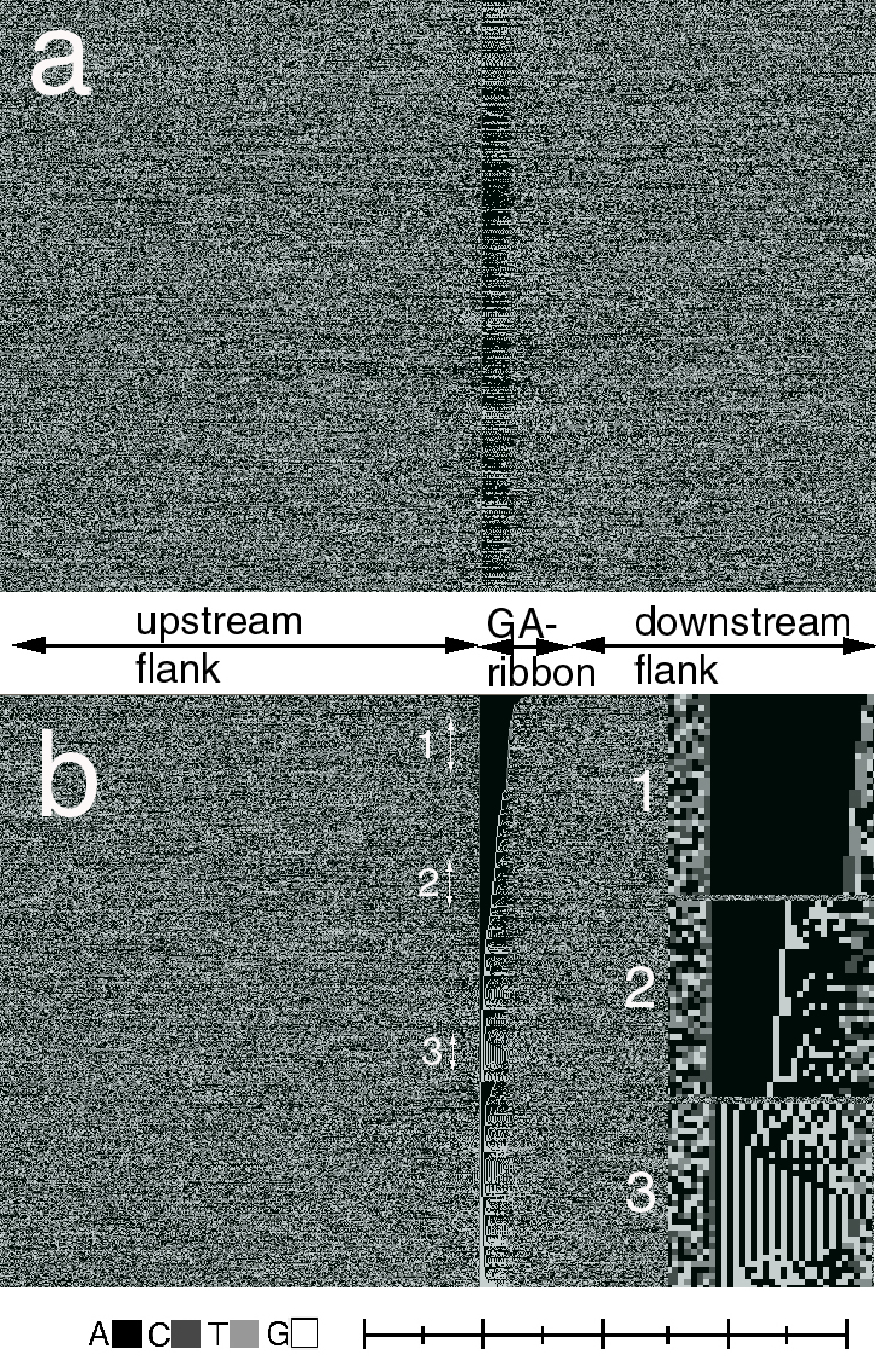

Supplement: Figure S4 — GPxI of the first 492 GA-complexes of Drosophila melanogaster chr.X. (Scale: 50 (b)/division) a. GPxI of the GA-complexes in their natural order aligned by the upstream start of the common GA-sequences. b. GPxI of the same GA-complexes sorted alphabetically. The right-hand insets show the 5× enlarged areas labeled 1, 2, and 3. 1: pure poly(A) GA-sequences. 2: poly(A) sequences ending in G. 3: one of the many examples of poly(GA) sequences. (9.89 MB TIF) [file pone.0004701.s004.tif]
