# Supplementary material for: Outline of a Genome Navigation System Based on the Properties of GA-Sequences and Their Flanks
Source: PLoS One. 2009 Mar 9;4(3):e4701. doi: 10.1371/journal.pone.0004701 (PMC2651618; doi:10.1371/journal.pone.0004701)
Supplement: Appendix S1 — The genome pixel image (GPxI). I introduce and apply here a novel method to represent DNA sequences. It turns relationships between sequences into visible patterns by representing the DNA sequences as gray-tone images called ‘genome pixel images’ (GPxI). The method is both sensitive and intuitive as it takes advantage of the exceptional ability of the human visual sense to detect patterns in images. (0.03 MB DOC) [file pone.0004701.s005.doc]

# APPENDIX S1

## The genome pixel image (GPxI)

The method assigns to each base arbitrarily a gray-tone value (see Fig.S1a) and transforms the consecutive bases of a DNA sequence into a continuous line of pixels of these gray-values. Whenever the line reaches the edge of the image area, it wraps around like any other text would, and continues at the beginning of the next line immediately underneath. For example, the GPxI of a computer-constructed, random DNA sequence appears as the featureless dot-pattern shown in Figure S1b.

## Examples of GPxIs and their interpretations

Figure S2 shows the GPxI of the first 150 Kb of the human X chromosome (Fig.S2a). While the size of 150 Kb is already too large for many applications of the traditional alignment methods, the striking patterns visible in the GPxI image in near the 5’ end highlight immediately the exact location for candidates of repetitive sequences without any prior knowledge of any special properties of the sequences in this location. Furthermore, one can see immediately that these special sequences occur in 2 clusters separated by a large stretch of non-repetitive DNA. Their pseudo-repetitive character becomes obvious through the action of 2 consecutive magnifications shown of Figures S2b and S2c: The larger the magnification, the less the repetitions of any patterns become detectable.

The distinction between repetitive and pseudo-repetitive sequences can be tested by GPxIs in a much more objective way, too. Obviously, the appearance of any patterns depends on the width of the GPxI, as it determines which downstream part of a sequence is written directly below it. Given a series of truly repetitive motifs there will exist specific values for the GPxI-width where the motifs fall into perfect register and, thus, generate a pattern of vertical lines (see e.g. Main text, Fig.S4). As shown in Figure S3a, at a GPxI-width of 610 [b] the obliquely striped patterns shown in Fig.S2a seem to turn into vertical lines (marked as ‘1’ in Figure S3). However, as shown by the magnified inset at the right hand side, the vertical lines are not perfect. Instead, they fall into 3 groups shifted out of register by 2 insertions. Furthermore, they are interrupted by numerous point mutations that appear as differently colored pixels within many vertical lines. Both properties identify them not only as pseudo-repeats, but also identify the causes of their differences.

The method of changing the width of the GPxI may also bring out the existence of otherwise easily overlooked relationships. For example, the domains labeled as ‘2’ and ‘3’ in Figure S3 may appear rather unstructured and, thus, unrelated at a GPxI-width of 568 [b], whereas pseudo-repetitive patterns become clearly visible at 551 [b] GPxI-width.

In summary, the method offers 4 important advantages over the traditional homology-based methods.

(A). No prior knowledge or suspicion of any special relationships between the tested sequences is required.

(B) No special data-processing is required beyond the simple and fast reading of the sequences in question and their base-by-base translation into a line of gray-tone pixel.

(C) In contrast to traditional homology-based methods, which become increasingly cumbersome, time consuming, and difficult to interpret as the numbers and sizes of the tested sequences increase, the detection of patterns in the GPxI’s becomes even easier and more meaningful under the same circumstances.

(D) Patterns and, thus, relationships between sequences remain easily detectable, even when mutations and other scrambling and distorting influences on the sequences may have randomized them and, thus, reduced the possibilities to demonstrate them mathematically to almost nil.

FIGURE LEGENDS

Figure S1.

**Basic principle of the 'genome pixel image' (GPxI) method.** (Scale: 50[b]/division)

a. Assignment of a pixel value to each base.

b. Creation of a pixel image by writing the sequence of a DNA file from left to right and top to bottom while expressing each base as a single pixel with the assigned gray-value. Whenever the pixel line has reached the edge of the image (= GPxI-width), it wraps around and continues on the left and 1 pixel diameter down. The GPxI shown in panel b represents a computer-constructed, random DNA file.

Figure S2.

**GPxI of the first 150 Kb of the human X chromosome (Un-sequenced portions are omitted).** (Scales: 50[b]/division)

a. The appearance of several pseudo-repetitive sequences as various, seemingly repetitive patterns. The appearance of identical repetition vanishes with increasing magnification of the GP demonstrating the power of the human visual sense to still detect rules and relationships between DNA sequences even after mutations and variations have obliterated them to a large degree..

b. Enlargement of the portion of the GPxI within the black frame in panel a.

c. Enlargement of the portion of the GPxI within the black frame in panel b.

Figure S3.

**Effect of GPxI-width on pattern appearance and recognition on a portion of the GPxI of Figure S2.** The numbers 1,2,and 3 indicate the same domains on each panel. Enlargments of these domains are shown on the right hand side.(Scale: 50[b]/division)

a. GPxI-width = 610 [b]. The pattern at '1' turns vertical but, as shown by the enlargement, contains deviations in the form of 2 shifts (=insertions) and single deviant pixels (=point mutations).

b. GPxI-width = 568 [b]. The domains '2' and '3' appear almost random.

c. GPxI-width = 551 [b]. Domain '2' shows a clear periodicity with few deviations. Domain '3'' shows pseudo-repetitive patterns.
