# Supplementary material for: Outline of a Genome Navigation System Based on the Properties of GA-Sequences and Their Flanks
Source: PLoS One. 2009 Mar 9;4(3):e4701. doi: 10.1371/journal.pone.0004701 (PMC2651618; doi:10.1371/journal.pone.0004701)
Supplement: Appendix S2 — GPxI of the common GA-sequences of Drosophila melanogaster, chr.X. Many of the supporting experiments in this article about vertebrate genomes were quoted from data about this non-vertebrate organism. In order to facilitate the comparison for experts in the field of Drosophila, the Appendix shows the GPxI of the X chromosome and the complete list of common GA-sequences. (0.44 MB DOC) [file pone.0004701.s006.doc]

**APPENDIX S2**

# List and GPxI of the common GA-sequences of Drosophila melanogaster chr. X (0 - 8 Mb)

Figure S4.

GPxI of the first 492 GA-complexes of Drosophila melanogaster chr.X.

(Scale: 50 [b]/division)

1. GPxI of the GA-complexes in their natural order aligned by the upstream start of the common GA-sequences.
2. GPxI of the same GA-complexes sorted alphabetically. The right-hand insets show the 5x enlarged areas labeled 1, 2, and 3. 1: pure poly(A) GA-sequences. 2: poly(A) sequences ending in G. 3: one of the many examples of poly(GA) sequences.

# List of the common GA-sequences of Drosophila melanogaster chr. X (0 - 8 Mb)

| **position** | **GA-sequence** |
| --- | --- |
| 5498 | AAAAAAAAAAAAGGAAAGGAGGAAAAAC |
| 10997 | AAAAAAAAAAAAAAAAAAAAAAAAAAAAT |
| 11077 | AAAAAAAAAAAAAAAAAAAAAAAAAAC |
| 36502 | AAAAAAAAAAAAAAAAAAAAAC |
| 50076 | AAAAAAAAAAAAAAAAAAAAAT |
| 88469 | GAGAGGAGAAGGAGGAAGAAAAGAGAGT |
| 176542 | AGGAAGAAGAAGAGAGAGGAAAC |
| 211169 | AGGAGGGGGGAGGGGAGGGAGGGAGAT |
| 276803 | AAAAAAAAAAAAAAAAAAAAAAAC |
| 405232 | AGAGAGAGAGAAAGAGAGAGAGAGGGAGAGGGC |
| 425797 | GGGGAGGGGGAGGGGGAGGGGC |
| 499571 | AGGAGGAGGAGGAGGAGGAGGAGGAGT |
| 501877 | GGGAGAAAAAAGAGGAGGAGGAGC |
| 555580 | GAAAAAGAAAAAAGGGGAAGGT |
| 563662 | GAAAGAAAAGAAGAGAGAAAAT |
| 570419 | GAAGGGGAGGGGGGGAGGGGGGT |
| 574033 | GAAAGAGAGAGAGAGAGAGAGAGAGAT |
| 575115 | AAGGAGGGGGGGGAGGGAGAAC |
| 582511 | AAAAAAAAAAAAAAAAAAAAAAAAAAAAAAAAAT |
| 664769 | GAAAGAAAAGAAAAGAAGAGAAAGGGAAAT |
| 755500 | AAGGGGGAGGGGGGGGGGGGGAC |
| 790077 | GGAGGAGGGGGGGGGGGGGGGT |
| 820806 | AAAAAAAAAAAAAAAAAAAAAC |
| 861906 | AAAAAAAAAAAAAAAAAGAAAGAGAGC |
| 921516 | AAAAAAAAAAAAAAAAAAAAAAAAAAAAAAAAAAT |
| 940479 | GGGAGGGAAAGGGAAAGGGGGAGAGAGAGAT |
| 979621 | GAGAGAGAGAGAGAGAGAGAGGGAGGGT |
| 1007714 | GGGGGGGGGGGGAGGAGAGAGC |
| 1058949 | GGGAAAGGGAAAGGGAAAGGGAGC |
| 1079131 | AAGGGAAAAGAAAGGAGGGGGAT |
| 1089226 | GAGGAGGAGGAGGAGAAGGAGAAGGAT |
| 1106610 | GAGAGAGAGAGAGAGAGGGGAC |
| 1117695 | AAAAAAAAAAAAAAAAAAAAAC |
| 1133200 | GAGAGAGAGAGAGAGAGAGGAAAAGAGAGAGT |
| 1168796 | GGAGGAGGGGGGGGGGGAGAGGGAT |
| 1212605 | AAGAAGAAAAAGAAAAAGAAGGGAAAGGAC |
| 1220670 | AAAAAAAAAAAAAAAAAAAAAAAAAAGC |
| 1232211 | GAGAGAGAGAGAGAAAGAGAGGGC |
| 1242983 | GAGGAGGAGAAGGAGGAGAAGGAGAAGAAGGAGGAGAC |
| 1273057 | AAAAAGAGAGAAAAAGGGGAAC |
| 1343374 | AGAGAGAGAGAGAGAGAAAGGGGT |
| 1347992 | AGAGAAAGGAAAAGAAGGGGAAGC |
| 1413893 | AAGAGAGAGAGAGAGAGAGAGAC |
| 1418908 | AAAAAAAAAAAAGAAAAAAGAAAT |
| 1431082 | GGGAGGGAAAGGAAAGGAAAGGAGC |
| 1431572 | AAAAAAAAAAAAAAAAAAAAAAAAAAT |
| 1450155 | AAAAAAAAAAAAAAAAAAAAAC |
| 1465685 | GAGGAAGAGGAGGAAAAGAAGC |
| 1474632 | AAGAGAGAGAGAGAGAGAGAGAGAGAGGGAGAGGT |
| 1476801 | AGGAAGGAAGGAAGGAAGGAAGGAAGGAAGT |
| 1500516 | AAGGAGGAGGAGAAGGAGAAGAAC |
| 1604260 | GGAAGGAAGGGAAAGAAGGGAC |
| 1625861 | GAAAGAGGAGGAGGAGAGAGAGGAGC |
| 1625894 | GAAGAGAGGAAGGAAAGAAGAAGGGAAAGGC |
| 1658287 | AGAGAAGAGAAGAGAGGAGGAGT |
| 1761974 | GGAGGAGGAGAAGAAGGAGAGC |
| 1768446 | GGAGGAGGAGGAGGAGGAGGAGC |
| 1771158 | GGAAGAAGAGGAGGAAGAGGAGT |
| 1793916 | AAAAAAAAAAAAAAAAAAAAAAAAAAAAGAAGAGT |
| 1821688 | AGAGAAAGAAAGGAGGAAAGAC |
| 1822325 | AAAAAAAAAAAAAAAAAAAAAC |
| 1827313 | GGGAGAGAGAGAGAGAGAGAGC |
| 1843117 | GGGAGAAAAGGGAGAGAGAGAGGGAGAGAGAGAGGT |
| 1843386 | GGAGAGAGGGAGAGAGAGAGAGAT |
| 1856770 | AAGAAAGAAAGAAAGAAAGAAAC |
| 1880159 | AAAAAGAGAGAAAGAGAAGAAAGC |
| 1974133 | AGAGGAAGAGGAGAAGGAGAAAAC |
| 2053141 | AAAAAAAAAAAAGGGAAAAAAGGT |
| 2113464 | GGAAAGGGGAGGAAGAGGAGAAGAT |
| 2138184 | GGAAAAAAAAAAAGAAAAAAGC |
| 2148579 | GGGGGGGGGGGGGGGGGAAGAGGAAGT |
| 2153563 | AGAGAGAGAGAGAGAGAGAGAGC |
| 2187725 | GGAGAGAGAGGGAGAGAGAGAGAGAGT |
| 2194832 | GAAAGAGAGAGAGAGAGAGAGAAAGAGAGAGAGAT |
| 2214041 | AAAAAAAAAAAAAAAAAAAAAC |
| 2238342 | GGAGAAGGAAAAGAAAAAAAAAAC |
| 2292369 | AAAAGGAGAAAGAAGAAAAAGC |
| 2307960 | AAAAAAAAAAGAAAGAGAAAAAGGT |
| 2318376 | AAAAAAAAAAAAAAAAAAAAAAAT |
| 2355237 | AAAAAAAGGAAAAAAAAAAAAC |
| 2391934 | AAAAAAAAAAAAAAAAAAAAAAT |
| 2405631 | AAAAAAAAAAGAAAAGAAAAAAC |
| 2411055 | AGAAGAAGAAGAAGAAGGAAAAAGC |
| 2487358 | AGAAAGAGAGAGAGAGAGGGAGAGAGC |
| 2511372 | GAGGAGGAGGAGGAGGAGGAGGAGGAC |
| 2527416 | AAGGGAAGAAGAAGAAAAGAAAAAAAAGAAAAAAT |
| 2529516 | GAAAAAAAAAAGAAAGAAAAGC |
| 2534102 | AAAAAAAAAAAAAAAAAAAAAAC |
| 2595063 | GGAGAGAGAGAGAGAGAGAGAGAGGGAC |
| 2614984 | GGGGGAGGGGGAGGGGGGGGGAGGC |
| 2616944 | GGGAGAGAGAGAGAGAGAGAGAC |
| 2628609 | AAAAAAAAAAAAAAAAAAAAAGAAC |
| 2637960 | GGAGAAAAAAAAAAGAAAAGAAGC |
| 2660059 | AAAAAAAAAAAAAAAAGAAAGC |
| 2671731 | GGGGAAAAAAAAAAAAAAAAAAAAAAAGT |
| 2674310 | AGAGAGAGAGAGAGAGAGAGAGC |
| 2689695 | AAAGGGGGAAAAAAAAAGAAAAC |
| 2709711 | GAGGAGGAGGAGGAAAGAAAAAC |
| 2712744 | AAAAAAGGGGGGGGGGGAAAAAAAAT |
| 2713701 | AGAAAAAAAAAAGAAGAAGGGGT |
| 2713918 | AAAAAAAAAAAAAAAGAAAAAT |
| 2715240 | AAAAAAAAAAAAAAAAAAAAAT |
| 2721002 | AAAAAAAAAAAAAAAAAAAAAAAT |
| 2723213 | AAAAAAAAGGAAGAAAAAAAAAGAAT |
| 2756610 | AAGGGGGGAGGAGGGGGGGGGGT |
| 2807184 | AGGAGGAGGAGGAGGAGGAGGAGC |
| 2813530 | AAAAAAAAAAAAAAAAAAAAAAAAT |
| 2815969 | AAAAAAAAAAAAAAAAAAAAAAAAT |
| 2828527 | AAAAAAAAAAAAAGAGAAAGAAGT |
| 2833726 | GGAGGAAAAAAAAAAGGGAAAC |
| 2838936 | GGAGGGGGGGGGGGGGGGGGGGGT |
| 2845355 | AAAAAAAAAAAAGGAGAAGAAAAC |
| 2856277 | AAAAAAAAAAAAAAAAAAAAAAAAAAAAAAAAAAAAAAAAAAAC |
| 2861323 | AGAGAGAGAGAGAGAGAGAGAGAGAGT |
| 2864426 | GAAAAGGAAAAAAAAGAAAAAAGAGGC |
| 2886527 | GAAAAAAAAGAAAGGAAAAGGAAAAT |
| 2889701 | AGAAGAAAAAAAAAAAGAAAAC |
| 2905293 | AAAAAAAAAAAAAAAAAAAAAAAAAAAAAAAC |
| 2911961 | AAAAAAAAAAAAGAAAAAAAAAAC |
| 2934462 | AAAAAAAAAAAAAAAAAAAAAT |
| 2939709 | GAGAAAAGGAGGGGGGGGGGGAC |
| 2948789 | GGGGGGAAAAAAAAAAGAAAAGC |
| 2950518 | AGAGAGAGAGAGAGAGAGAGAGGGAC |
| 2951343 | AAAAAAAAAAAGGAAAGAGAAGC |
| 2955698 | AAAAAAAAAAAAAAAAAAAAAAT |
| 2959772 | AAAAAAAAAAAAAAAAAAAAAAT |
| 2962724 | AAGGAGGAGAAGGAAGAAGAAAAAGAGGGAGC |
| 2963569 | AAAAAAAAAAAAAAAGGAAAAAT |
| 2977477 | GAAGGGGGGAGGGGGGGGGGGGT |
| 2986422 | GAGAGAGAGAGAGGGAGAGAAAC |
| 2996473 | AAAGAAAAAAAGAAAAAAAAAAT |
| 3002164 | GAAAAAAAGGGGAAAAGGGGAAAAAGC |
| 3008513 | GGGAGGAGGAGGAAGGGGGGGAGGGGGT |
| 3023163 | GAAAGAAAGAAAGAAAAGAAGAAAAAAAAGGAAAAAAC |
| 3035981 | AAAAAAAAAAAAAAAAAAAAAAAAAAAAAAAAAAAAT |
| 3037956 | GGAAAGAAGGGGGGGGAGGGGGGT |
| 3040871 | AAAAGAAAAAAAAAAAAAAAAT |
| 3042282 | AAGAGAGAGAGAGAGAGAGAAAGAGAGAGAGAT |
| 3048721 | AAAAAAAAAAAAAAGAAAAAAAAT |
| 3056826 | AAAAAAAGAAGAAAAAAAAAAAAAAC |
| 3085975 | AAAAAAAGAAAAAAAGAAAAAAAAAAAAGC |
| 3107971 | AAAAAAAAAAAAAAAAAAAAAAAGAAGT |
| 3109206 | AAAAAAAAAAAAAAAAAAAAAT |
| 3109754 | AAAAAAAAAAAAAAAAAAAAAAGAGGAAAAC |
| 3129961 | GGAAAGGGAAAAGGAAAGGGAAAAAGGAC |
| 3130501 | AAAAAAAAAAAAAAGAAAAGAGAAAAC |
| 3144184 | GAGAGAGAGAGAGAGGGGAAGAT |
| 3146744 | GAAGAAGAAGAGGAAGAGAGAAAGAGGAC |
| 3149435 | GAGAGAGAGAGAGAGAAAGAGAGAGGGAAAAGAC |
| 3154156 | AAAAAAAAAAAAAAAAAAAAAAAAAT |
| 3159259 | AAAGAAAAAAAAAAAAAAAAAAC |
| 3159796 | AGAGAGAAAGAGAGAGAGAGAGAAT |
| 3164067 | GAGAAGGAAGAAAGAAAAAAAAAC |
| 3169907 | AAAAAAAAAAAAAAAGAGAGAGAGAAT |
| 3171040 | AAAAAAAAAAAAAAAAAAAAAAAAAAGAC |
| 3187585 | AAAAAAAAAAAAAAAAAAAAAAT |
| 3191466 | AAAAAAGAAAGAAAGAAGGAAAAGT |
| 3193952 | GAAAAAGAAGAGAGAGAAAAAAT |
| 3194903 | AAAAAAAAAAAAAGAAGAAAAAAAAT |
| 3230263 | AGAAAAAAAAAAAAAAAAAAAC |
| 3234273 | AAAAAAAAAAGAAGAAAAAAAAC |
| 3234970 | GAAAGAAAAGAAAAGAAAAGAAAAGGAAGAAAAC |
| 3264048 | GAAAGAAAAAAAAAAAGAAAAAAGGC |
| 3264221 | AAAAAAAAAAAAAAAAAAGGGT |
| 3264666 | GAAAAAGAAAAAAAAAGAAAGAAAAAT |
| 3265832 | AAAAAAAAAAAAAAGAAAAAAGC |
| 3276751 | AAAAAAAAAAAAAAAAAAAAAAAAAAAAGC |
| 3278332 | AAAAAAAAAAAAAAAAAAAAAGAGAAAAAAAAGGT |
| 3278654 | AAGAAAAAAAAAAAAAAAGAAGAAAAAC |
| 3289719 | AAAAAAAAAAAAAAAAGAAAAAAAGAAGT |
| 3306266 | AAAAAAAAAGAAAAAGAAAAAAC |
| 3319707 | AAAAAAAAAAAAAAAAAAAAAAAAAAAT |
| 3327003 | AAAGGAAAGAAAAAAAAAGAAAAAAAC |
| 3339985 | AAAAAAAAAAAAAAAGAAAAAC |
| 3364934 | GGGAAGAAAAGAAAAAAAAAAAAC |
| 3372895 | AAAAAAAAAAAAAAAAAAAAGAGAGC |
| 3374096 | AAAAGGAAGGGGGGGGGGGGGGGGGGC |
| 3378141 | GGGAGAAGAAAAAAAAAAAAAAAAT |
| 3386673 | GAGAAAAAAAAAAGGGGAAAGC |
| 3388710 | AAAAAAAAAAAAAAAAAAAAAAAAT |
| 3389707 | AAAAAAAAAAAAAAAAAAAAAAAT |
| 3390252 | AAAAAAAAAAAAAAAAAAAAAAAAGT |
| 3401855 | AAAAAAAAAAAAAGAAAAGGAAAAT |
| 3402501 | AAAAAAAAAAAAAGAAAGGAAGAAAC |
| 3405299 | GAAAAAAAAAGAAGAAAAAAGAAC |
| 3406581 | AGAAAGAGAGAGAGAGAGAGAGGGC |
| 3412499 | AAAAAAAAAGGAGGGGGGGGGGGGC |
| 3412764 | AAAAAAAAAAAAGAAGAAGAAGAAT |
| 3416687 | GAAGAAAGGGGAGGGGGGGGGGGGGT |
| 3439921 | GAAGAAAAAAAAAAGAAAAAAAGAAAAAC |
| 3445013 | AAAAAAAAAAAAAAAAAAAAAAC |
| 3468257 | AAAAAAAAAAAAAAAAAAAAAAGT |
| 3500047 | AAAAAAAAAAAAAAAAAGAAAAAAAGGAAGGAAAT |
| 3512826 | GAGAGAGGGAGAGAGAGAGAGC |
| 3523205 | AAAGAAAGAGAGAGAGAGAGAGC |
| 3537573 | AGAGAGAGAGAGAGAGAGAGAGAGAGAGT |
| 3549937 | AAAAAAAAAAAAAAGAGGAAGC |
| 3550115 | AAAAAAAAAAAAAAAAAAAAAAAAAT |
| 3557041 | AAAAAAAAAAAAAAAAAAAAAAC |
| 3565866 | AAGAGGAAGAGAAAAAAAAAGGGT |
| 3571748 | AAAAAAAAAAAAAAAAAGAAAAC |
| 3586456 | GAGAGAGGGAGGGAGAGAGAGC |
| 3605791 | AAAAAGAAAGAAAAAAAAGGAGC |
| 3605815 | AAAAAGAAAGAAAAAAAAGGAGC |
| 3613891 | GAAAGAAAGAAGGAGAAAAAAC |
| 3644310 | AAAAAAAAAAAAAAAAAAAAAAT |
| 3677560 | AGAGAAAGAGGAAGGAGAGGAGGAAT |
| 3683054 | GAGAGAAGAAAGAGAGAGAGAGAGAGAGAGAGAGAC |
| 3725560 | GGAGAAGGGAGGAGGGGGGGGAGGAAAC |
| 3746359 | AAAAAAAAGAGGGGAAAAAAAAAGAAAAAAGGC |
| 3813577 | AAAAAAAAAAAAAAAAGGGGAGC |
| 3816820 | AGAAAAAAAAAAAAAAAAAAGGT |
| 3841209 | AGAGAAGAGAAAGGGGGAGAAAGT |
| 3841269 | GGAGAGAAAGAGAAAGAGAGAGGC |
| 3841580 | AAAAAAAGGGAAGAAGAAAAAGC |
| 3844284 | AAAAAAAAAAGAAAAAAAAAAGT |
| 3845351 | AAGGAAGAAGAAGAAGAAGAAGAGGAGC |
| 3850034 | AGAAAAAAAAAAGAAGAAGAAT |
| 3858006 | GAAAAAAAAGAGAAAAAAAGGAAGGAAGC |
| 3874615 | AAAAAAAAAAAAGAAAGGGGGT |
| 3875426 | GAGAAAGAGAAGAGGAGAGAGAAAAAAAGC |
| 3879058 | AAAAAAAAAAAAGAAAAAGAAGT |
| 3883698 | AAAAAAAAAAAAAAAAAAAAAT |
| 3901620 | AGAAAAAAAAAAGAAGAAAAAAAAAAGGGAAAGGAGC |
| 3909952 | GGGGAAGGGGAAAAAGGGGGAGAC |
| 3916659 | AAAAAAAAAAGAAGAGAAAGAGAGT |
| 3917145 | AGAGAGAGAGAGAGAGAGAGAGAGAGAGAGAGAGAT |
| 3924042 | GAAAGAAAAAAAAAAGAAAAAAAAAAAT |
| 3976965 | AGAAGAAAAAGGAAGAAAAGGAAAC |
| 3993873 | AAAAAAAAAAAAAGGAGAAAGGGAGGT |
| 4005616 | GAAAGAAAGGAGGGGGGGGGGGT |
| 4016119 | AAGAGAAAAAAAAAGGAAGGGGC |
| 4025847 | AAAAAAAAAAAAAAAAAAAAAAC |
| 4030965 | AAAAAAAAAAAAGAAAGGGAAAAAAC |
| 4038306 | AAAAAAAAAAAGAAAAAAAAAAAC |
| 4107186 | GAAGGAGGAGGAGGAGGAGGAGT |
| 4134835 | GAAAAAAAAAAAGAAAAGAAAAGGAT |
| 4186699 | AGAAAAAGAGAGAGAGAGAGAGC |
| 4189444 | AGAAAGGAAAAAAAAAAAAAGAAAC |
| 4189875 | GAGAAAAGAAGGAGGAGGAGGGT |
| 4197411 | AAAGAGGAGGAAAAGAAGGAGC |
| 4233805 | AAAAAAAAAAAAAAAAAAAAAAAC |
| 4238807 | AAAAAAAAAAAAAAAAAAAAAAAAC |
| 4251867 | AAAAAAGAAAAAAAAAAAAAAAC |
| 4255611 | GGGGGGGGGGGGGGAGAGGGGGT |
| 4259440 | GAGAGAGGGAGAGAGAGGGAGC |
| 4268831 | AAAAGGAGGGGGGGGGGGGGAC |
| 4272538 | AAAAAAAGAAGAAAGGAAAGAAAAT |
| 4277001 | GGAGAAAGAAAGAGAAAAAAGAC |
| 4282424 | AAAAAAAAGGAAAAGAAAAGGC |
| 4283459 | AAAAAAAAGAAAAAAAAAAAAAC |
| 4286312 | AAAAAAAAAAAAAAAAAAAGAGAAAAAGC |
| 4289184 | GAAGGGAGGGAGGGAGGGGGGGGGC |
| 4293691 | GAAAAAAAGGAAGAAAAAAAAGC |
| 4294890 | AAAAAAAAAAAAAAAGAAGAAAAAAAAT |
| 4297956 | AAAAAAAAAAAAAAAAAAAAAAT |
| 4304228 | AAAAAAAAAAAAAAAAAAAAAAAAAGGAAGAAGAC |
| 4304909 | GGAAGGGGGGGGAGGGAGGGAGGGC |
| 4327172 | GAAGGGGGGGAAGAAAGGAGGGT |
| 4408193 | AAAAAAAAAAAAAAAAAAAAGC |
| 4463701 | AAAAAAAAAAAGAAAAAAAAAAC |
| 4516585 | AGAGAGGGAGGGGAGAAAAAGAC |
| 4526979 | AGAGAGAAAAAGAGAAAGAGAGAGAGAGAGAGAC |
| 4528467 | AGAGAGAGAGAGAGAGAGAGAGAGAGC |
| 4548916 | GGAGAGAGAGAGAGGGGGGGGGT |
| 4558354 | AAAAAAAAAAAAAAAAAAAAAAAAC |
| 4597996 | GGAAAAGAGGGGGGGGGGGGGAT |
| 4602254 | AAAAAGAGAGAGAGAGAGGGAGAGAGC |
| 4616988 | AGAAAGAGAGAGAGAGAGAGAGGC |
| 4631213 | GAAGAGGGGAAAGGGAGAGAAGT |
| 4691979 | AAAGAGAGAGAGGGAGAAAAAAAAAAC |
| 4695331 | AAAAGGGAAAAAAAAGGGAAAAAAAGAAAAGGAGT |
| 4703725 | GAAGGGGGGGGGGGGGGGGGGT |
| 4718184 | AGAAAGGGAAAGAGAGAGAGAGAGAGAAAGAGAC |
| 4728514 | AAAAAAAGAGAAAAAAAAAAGAAC |
| 4801506 | AAAGGGGGAAGAGGGGGGGGGGGT |
| 4810848 | AAAAGAAAAAAAAAAAAAAAAAAAAAC |
| 4843517 | GAAAAAAAAAAGAAAAAAAAAT |
| 4853241 | GGGGAAAAAAAAAGGGGGGGAAAAAT |
| 4854525 | GGGGGGAGGGGGGGAGAGGAGGGGGAT |
| 4874912 | AAAAAAAAAAAAAGAAGGAAGAT |
| 4878740 | AAAAGAAAAAGAAAAAAAAAAC |
| 4888708 | GGAAAAAGAAAAAGAAAAGAGT |
| 4890517 | GAAAGAGAGAGAGAGAGAGAGAGAGAC |
| 4892576 | AAAAAAAAAAAAAAAAAAGGAAGAT |
| 4893658 | AAAAAAAAAAAAGAAAAAAAAAAC |
| 4897613 | AAAAAAAAAAAAAAGAAAAAAAAAAGGAT |
| 4907168 | GAAAAAAAAGAAAAAAAAAAAC |
| 4926135 | AAAAAAAAAAAAAAAAAAAAAAAAAAAAC |
| 4928128 | AAAAAAAAAAAGGAAAAAAAAAAAC |
| 4929655 | AAAAAAAAAAAAAGAAAAAAAAAC |
| 4941578 | AGGAGGAGAAGAAGAAGAAGAAGT |
| 4948445 | AAAAAAAAAAAAAAAAAAAAAT |
| 4951162 | AAAAAAAAGGGGGGGGGGGAAT |
| 4952433 | GAGAGAGAGAGAGAGAGAGAGAGAGAGAGGGGGGAGC |
| 4964519 | AAAAAAAAAAAAAAAAAAAAAAAAT |
| 4971253 | AAAAAAAAAAAAAAGAAAAGGAAGAGAAAAT |
| 4979922 | AAAAAAAAAGAAAAAAAGAAAAAAAGAAAAAAAAGAAAAAAAAGAAAAAAAAAAGAAAAAAT |
| 4984599 | GAAAAGAGAGAGAGAGAGAGAGAGAGAGAT |
| 4994186 | AAAAGGGGGAAGGGGGGGGGGGGC |
| 4998430 | AAAAAAAAAAAAAAAAAAAAAAAGAAAT |
| 4998595 | AAAAAAAAAAAGAAAGAAAGGGC |
| 5007167 | AAAGGAAAGAAAAAGAAAAAAAAGAAAAAAT |
| 5010961 | AAAAAAAAAAAAGGGGAAAAAAAT |
| 5012406 | AAAAAAAAAGAAAAAAAAAAAC |
| 5013666 | GAAGAGGAGGAGGAGGAGGAAGAGGAGC |
| 5014338 | AAGAAGAAAAAAAAAAAGGAAAC |
| 5014394 | AGAAGAAAAAAAAAAAAAAAAAT |
| 5015768 | AGGGAAAGAAAGGAGGAAAAGC |
| 5024532 | AAAGAGAAAAGGAAAGAAAAGAAAGAAAAAAC |
| 5029550 | GGGAAAAGAGAGAGAGAGAGAGAGGGAGAGGT |
| 5030317 | AGAGAGAGAGAGAGAGAGAAGAGT |
| 5036802 | AAAAAAAAAAAAAAGAGAAAAAAAC |
| 5037720 | AAAAAAAAAGGAAGAAAAAAAAGT |
| 5043909 | GAGGGGGGGGGGGGGGAGAAGAAAT |
| 5054534 | AAAAAAGAGAGGAAGGGAAAAGGAGAAAT |
| 5067345 | AAAAAAAAAAGAAAAGAAAAGAAAGGGAAAT |
| 5135837 | AAAAAAAAAAAAAAAAGAAAAAAAAAGGC |
| 5142745 | AAAAAAAAAAAAAAAAAAGAAAAC |
| 5144751 | AAAAAGGAAAAAAAAAAAAAAAAC |
| 5149255 | GGGAAGAGAAGAAAGAAAAAAAAAAGGAAAAT |
| 5152685 | AAAAAAAAAAAAAGAAAAAAAAAAAC |
| 5171943 | AAAAAAAAAAAAAAAAAAAAAAT |
| 5182819 | GGGGGGAGGAGAGGAGAGGAGAGGAGAGGAGC |
| 5192387 | AAAAAAAAAAAAAAAAAAAAAAAC |
| 5201720 | GGAGAGAAAGAGAGAGGGAGAGAGAGAGAGT |
| 5224677 | AGGAGGAGGAGGAGGAAGAGGC |
| 5232861 | AGAAGAAAAAAAGGAAAAAAAAAAGAAAAAGAAAAGAAC |
| 5274481 | GGAGGGGAGGGGGGGGGAGAGT |
| 5285004 | AAAAAAAAAGAAAAAAAAAAAAAAAAC |
| 5291844 | AAAAAAAAAAAAAAAAAAAAAC |
| 5293661 | GAAAGGGGGGAAAGGAGGAAAGGAGGAAAAGC |
| 5299543 | AAAAAAAAAAAGAAAAAAAAAAAAAC |
| 5301497 | AAAAAAAAAAAAAGGGGGGAAT |
| 5302732 | AAAAAAAAAAAAAAAAAAAAAAC |
| 5307411 | AAAAAAAAAAGAAAAAAAAAAT |
| 5312246 | AAAGAAAAAAAAAAAAAAAAAAAC |
| 5313118 | AAGAGAGAAAGAGAGAGAGAGGGGGGAAGAAAGAAAC |
| 5335780 | AAAAAAAAAAAAAAAAAGAAAAAAAAAT |
| 5337746 | AGAGAGAGAAAAAAAAAAGAAAT |
| 5372019 | GGGGAAAGGGGAGAAGAGGGGGC |
| 5390275 | GAAAGAGAGAGAGAAAGAGAGAGAGAGAGGGGGGGGGGT |
| 5407263 | AGGAAAAAAGGAAAAAAAAAAAAAGAC |
| 5424684 | GGGGGGGGGGGGGGAGGGAGGT |
| 5440229 | GGGAAAAGAAGAGAAAAAAAAGT |
| 5459768 | GGGGAGAGAGAGAGGGGAAAGAAAGGAAAAC |
| 5469025 | AGAGAGAGAGAGAGAGAGAGAT |
| 5487662 | GGGGGGGGGGGGGGAGGGGGGGT |
| 5505114 | GGAGAAGGAGAAGGAAAAGGAGAAGC |
| 5515042 | GGAGGGGGGAGGGGGGGAGGGGGC |
| 5569621 | AAAAAAAAAAAAAAAAAAAAAAAAT |
| 5573071 | AAAGAGAAGAAAGGGAAAAAGC |
| 5608351 | GAGAGGGAGAGGAAGAGGGAGAGGGAGAGGAGC |
| 5640947 | AAAAAAAAAAAAAAAGAGAAGAT |
| 5643937 | GGAGGGAGGAAGGGGGGGGGGT |
| 5644982 | AAAAAAAAAGAAAAGAAAAGAAAAAAAAAAGGGAT |
| 5651031 | GGAGAAAGAAAAAAAAAAAAAAAAC |
| 5656352 | GGGAAGAGAGGGGAGGGGGGAGAC |
| 5686713 | GGAGAGGGAGGGGAGGGGAGGGGT |
| 5689320 | AAAAAAAAAAAAAGAGAGAGAGAGAC |
| 5737467 | AGAGAGAGAGAGAAAAAGAGGGAGAGC |
| 5749269 | GAAGAAGAAAAGAGAAAAAAAAAC |
| 5756291 | GAGAGAGAGAGAGAGGAAGAGAGC |
| 5771059 | AAAAAAAAAAAAAAAGAAAAAAAAAAAC |
| 5834478 | AAAAAAAAAGAAAGAAAGAAAAAAAAAAAC |
| 5958096 | AAAGAGAGAAAGAGAGAGAGAT |
| 5959901 | GAGAGAGAGAGAGAGAAGAGAGAAGT |
| 5976596 | AAAAGAGGGGAAAAAAAAGAAGAAAAT |
| 5978377 | AAAAAAAAAAAGGAAGAAAAAAAAAAC |
| 6046035 | GAAAGAAGAAAAAAAAAAAAAGAAAAC |
| 6051514 | AAAAAAAAAAGAAAAAAGAAAAGAAAAAGGC |
| 6102394 | AAAAAAAAAAAAAAGAAAAGAAAAAAAAT |
| 6105294 | AAAGAAGAAGAGAAGAGGAAGAAAC |
| 6123968 | GAAAAAAGAAAAGAGAGGAAGT |
| 6147672 | GAGAAAAAAGGAAGGAAGGAGGGGGT |
| 6148368 | GAGAGAGAGAGAGAGAGAGGGAGAGAGC |
| 6177923 | GAGGGAAAAGAGAAAAAAAAAAC |
| 6217117 | AGAGAGAGAGGGAGAGAGGAAAAGAGAGGGAAGGAGAGGC |
| 6218674 | GAAAAAAAAAAAGAAAAAAAAT |
| 6226029 | AAGAGAGAAAGAGAGAGAGAGAGAGAGAGT |
| 6228796 | GAAGAAAAGGAGGGAAGGAGGAAGGAT |
| 6235084 | AAAAAAAAAAAAAAAAAAAAAAAAAAAGAT |
| 6246568 | AAAAAGGGGGAAAAAAAAAAAC |
| 6249065 | AAAAAAAAAAGAAGAAGAAGAAGAAGAC |
| 6276234 | AAAAAAAAAAAAAAAAAGAAAAC |
| 6276478 | AAAAAAAAAAAAAAAAAAGGAT |
| 6277169 | AAAAAAAAAAGAAAGAGGGGAGAT |
| 6277520 | GAGGAAAAAAAAAAAAGGGGAAAC |
| 6297024 | AAGAAAAAAAAAAAAAAGGAGC |
| 6298119 | GGAAAGGAGAGAAAGAGAGAGAGAGAGAGAGAGAGAGAGC |
| 6320784 | AAAAAAAAAAAAAAAAAAAAAAAGAAAAGT |
| 6330085 | AAAAAAAAAAAAAAAAAAAAAAAAAAT |
| 6333073 | GGAAAGGAAAAAAGAAAAGAAAGGAAGT |
| 6343143 | GGGGGGGGGGGGGGGGAAAAGAAC |
| 6344975 | GAGGGAAGGGAGGGGGGGGGGT |
| 6346043 | GAAAAAAAAAGAAAAAAGAAAAAC |
| 6346602 | GAGAGAGAGAGAGAGAGAGGGAGAGAT |
| 6350067 | AAAAGAGAAGGAAAGGGAAGAAAT |
| 6351031 | GGGAGAGGGGGGGGGGGGGGGT |
| 6352972 | AAAAAAAAAAAAAAAAAAAAAGGAAAAAAAAGAAGGGGAAAC |
| 6357514 | AAAAAAAAAAAAAGAAAAAAGT |
| 6362588 | GAAGGAGAAAGAGAAGGAGAAGGGT |
| 6411319 | AAAAAAAAAAAAGAAAAGAGAAAAAGT |
| 6415452 | GAAGGAGGAAAAGGAAGAGGAGGAAAGC |
| 6429028 | AAAAAAAAAAAAAAGGAAAGGC |
| 6434099 | AAAAAAAAAAAGAAGAAAAAAAAAT |
| 6446496 | AAAAAAAAAAAGAAAAAAAAAAC |
| 6458724 | AGAAAGAAGAAGAAAAAAAAAT |
| 6467725 | AAAAAAAAAAAAAAAAAAAAAAAT |
| 6477913 | AAAAAAAAAAAAAAAAAAAAAAC |
| 6480872 | AAAGAGGAGAAGAAAAGAAGAAGC |
| 6481820 | AAAGGGGGGAAGGGGGGAAAGGGGT |
| 6483881 | GAAAAGAAAAGAAAAGAAAAAGC |
| 6490545 | AAAAAAAAAAAAAAAAAAAAAAAAAAAAAAAGAAC |
| 6516520 | GAAAAAAAAAGGGAAAAGAGAGAAC |
| 6565291 | AAAAAAAAAAAAGAAAAAAAAGAAAAC |
| 6590192 | AAAAAAAAAAAAAGAGAAAGAC |
| 6661995 | AAGAAAAGAAAAGGAAAAGAAC |
| 6690992 | GGAAAGGGGGAGGAGAAGAAGAAGAAGAGGAGGT |
| 6702804 | GGAGAAGGAGAAGAGGAGAGGAAAGC |
| 6727211 | AAAGGGGGAAAGGGGGGGGGGAGC |
| 6740602 | AAAAAAAAAAAAAAAAAAAAAAAAAAAGAGAAAAC |
| 6747901 | GGAAGGGGGGGGAGGAGGGGGAAAAAT |
| 6771405 | GGGGGGGGGGGGGGGGGGGGGGGAAAGAGT |
| 6776344 | AAAAAAAAAAAAAAAAAAAAAC |
| 6778848 | GGAAGAGGAAAGAAGAAAAAAAC |
| 6821432 | GGGGAAGAGAGAGAGAGAGAAAGAAAAGAGAGAAAAAGGGGGT |
| 6826537 | GAGAGAGAGAGAGAGAGAGAGAGAGAGAGC |
| 6867771 | AAAAAAAAAAAAAAAAAAAAAAAAAAC |
| 6872355 | AAGGAAAAAGGAGGAAGGAAAAAAAT |
| 6873382 | GAGGAGGAGGGGGGAGGGGGAAGGGGGAGT |
| 6896716 | GAAAGGAAAAGGGAAAGGAAAGGGT |
| 6926603 | AAAAAAAAGAAAAAAAAAAAGAGT |
| 6927210 | AAAAAAAAAAGGGGAAAGAAGAAGC |
| 6931823 | GGAGAAGGGGGAAGAAGAGGAGC |
| 6934760 | GGGAGAGAGAGAGAGAAGGAGT |
| 6952403 | AAAAAAAAAGAAAAAAAAGGAAGAAAT |
| 7010415 | AAGAAAAAAAAAAAAAAAAAAAAC |
| 7015783 | AGAAAAAAAAAAAAAAAAAAAC |
| 7024505 | AAAAAAAGAAAGAGAGAAAAAAC |
| 7052191 | AAAAAAAAAAAAAAAAAAAAAAAAAT |
| 7056968 | GAAGAAGAAGAAGAAGAAGAGAAGGGC |
| 7062567 | GGAGAAAGAGAGAGAGAGAGAGAGAGAGAGAGAGAGAGT |
| 7072463 | AAGAGAAGAGGAGAGGAGAGAAAGC |
| 7073629 | AGGAAAGAAAAAAAAAAAGGAAAAGT |
| 7101097 | AGAGAGAGAGAGAGAGAGAGAGC |
| 7106092 | AAAAAAAAAAAAAAAAAAAAAAAAAGAAGT |
| 7113046 | AGAGGGAAAAGGGGGAAGAAGGAT |
| 7113212 | AAAAAAAAAAAAAAAAAAGAAGAAGC |
| 7131292 | GAAAGGAAGAAGAAGAAGAAGC |
| 7139769 | GGAAGAGGAAAAAAAAGGAGGC |
| 7140353 | AAAAGGGGAGGGGGGGGGGGGGGT |
| 7170396 | AAAAAAAAAAAAAAAAAAAAAC |
| 7186512 | AAAAAAAAAAAAAAAAAGAAAC |
| 7220178 | AAAAAGAGAGAGAGAAAAAAAAAAAAC |
| 7276342 | AAAAAAAAAAAAAAAAAAAAAAAAAC |
| 7308625 | AAAGGAAAGAAAAAAAAAGGGGT |
| 7316963 | AGAAAGAAAGGGAAGGGGGGGT |
| 7319778 | AAAAAAAAAAAAAAAAAGGGAAAAGAAT |
| 7329957 | GGGGAGGGGGAGGGGGGGGGGT |
| 7375000 | AGAGAAAGAGAGAGAGAGAGAGAGAGAGAGAAAGAC |
| 7379411 | AAAAAAAAAAAAAAAGAAAAAAT |
| 7404627 | GAAGAAGAAAGGAAAGGAAAGAGAT |
| 7410685 | GAAGAGAGAGAGAGAGAGAGAGAAAGAGAGAAC |
| 7426685 | AAGAAGAAGAAAAGAGAAAGGGGAAGT |
| 7428755 | AAAAAAAAAGGGGGAAAAAAAAC |
| 7453463 | AAAAAAAAGAAAGAGAGAGGGAGAT |
| 7453488 | AGAGGGGAGAAGAAAAAAGAAAAAT |
| 7460925 | AAAAAAAAAGAAGAAGAAGAAGAAAAAAC |
| 7466704 | GAAGGAAAAAAAAAGAAAAAAC |
| 7480186 | AAAAAAAAAAAAAGAAGAAAAGT |
| 7503827 | AAAAAAAAAAAAAAAAAAGAAAAGAGAGT |
| 7535674 | GGGGGAGAAGGGGGAGAAGAGAGC |
| 7536218 | AAGAAAAAGAAGAAGAAAAGAGAAAAT |
| 7538178 | AAAAGAAAGAAAAAAAAAAAAC |
| 7540785 | AAAAAAAAAAAAAAAAAAAAAAAAAAAAAAAAAT |
| 7556737 | AAAAAAAAAAAAAAAAAGAAAAGC |
| 7580365 | GAAGAAGAAGAAGAAGAGGAAC |
| 7592222 | AGAAGAGAGAGAGAGAGAGAGAGC |
| 7607496 | AAGAAAGAGAGAGAGAGAAGGAGAGAGT |
| 7662032 | GAGGAGGGGGGGGGGGGGGAAT |
| 7672967 | AAAAAAAAAAAAAAAGAAGAAGGGGAAGGAGAAAC |
| 7684530 | AAAAAAAAAAAAAAAAAAAAAAAT |
| 7796745 | AGAGAGAGGGAGAGAGAGAAAGAT |
| 7813921 | AAAAAAAAAAAGAAAAGAAGAAAAAAAGAAAAGC |
| 7816172 | GAAGAGGAGAGGGGGGGGGGGT |
| 7816197 | GGAGAGAAAGGAGAAAGGAGAGT |
| 7852053 | AAAAAAAAAAAGAAAGAAAAAAAAAAAAAAC |
| 7853736 | GGGAAGGGAAGGGGAAGGGGAAC |
| 7884226 | AGAGAGAGAGAGAGAAAGAGAT |
| 7886834 | GAGAGAGAGAGAGAGAGAGAAGT |
| 7925443 | AAGAGAGAGAGAGAGAGAGAGAGAGAAC |
| 7990817 | AGAGGAGGAGGAGGAGGAGAAGGAGGAAGGT |
